# A novel microRNA, novel-m009C, regulates methamphetamine rewarding effects

**DOI:** 10.1038/s41380-022-01651-2

**Published:** 2022-06-17

**Authors:** Li Zhu, Feifei Wu, Zhilan Yan, Lijun He, Shufei Wang, Haohao Hu, Eyleen L. K. Goh, Yingjie Zhu, Fanglin Guan, Teng Chen

**Affiliations:** 1 College of Forensic Medicine, Xi’an Jiaotong University Health Science Center, Xi’an, 710061 Shaanxi PR China; 2 The Key Laboratory of Health Ministry for Forensic Science, Xi’an Jiaotong University, Xi’an, 710061 Shaanxi PR China; 3 Neuroscience and Mental Health Faculty, Lee Kong Chian School of Medicine, Nanyang Technological University, 308232 Singapore, Singapore; 4 Shenzhen Key Laboratory of Drug Addiction, CAS Key Laboratory of Brain Connectome and Manipulation, the Brain Cognition and Brain Disease Institute (BCBDI), Shenzhen Institutes of Advanced Technology, Chinese Academy of Sciences, Shenzhen, 518055 PR China; 5 Shenzhen-Hong Kong Institute of Brain Science-Shenzhen Fundamental Research Institutions, Shenzhen, 518055 PR China

**Keywords:** Addiction, Diagnostic markers

## Abstract

Methamphetamine (METH) is a widely abused psychostimulant, whose hyper-rewarding property is believed to underlie its addictive effect, but the molecular mechanism regulating this effect remains unclear. We previously reported that decreased expression of a novel microRNA (miRNA), novel-m009C, is implicated in the regulation of METH hyperlocomotion. Here, we found that novel-m009C may be homologous to hsa-miR-604. Its expression is consistently downregulated in the nucleus accumbens (NAc) of mice when exposed to METH and cocaine, whereas significant alterations in novel-m009C expression were not observed in the NAc of mice subjected to other rewarding and psychiatric stimuli, such as sucrose, morphine and MK-801. We further found the substantial reduction in novel-m009C expression may be regulated by both dopamine receptor D1 (D1R) and D2 (D2R). Increasing novel-m009C levels in the NAc attenuated METH-induced conditioned place preference (CPP) and hyperlocomotion, whereas inhibiting novel-m009C expression in the NAc enhanced these effects but did not change the preference of mice for a natural reward, i.e., sucrose. These effects may involve targeting of genes important for the synaptic transmission, such as Grin1 (NMDAR subunit 1). Our findings demonstrate an important role for NAc novel-m009C in regulating METH reward, reveal a novel molecular regulator of the actions of METH on brain reward circuitries and provide a new strategy for treating METH addiction based on the modulation of small non-coding RNAs.

## Introduction

Methamphetamine (METH) is a widely abused drug across the globe and is now the number one drug of concern in China [1, 2]. Evidence has revealed that METH exposure produces a strong euphoric effect by increasing extracellular dopamine levels [3–6]. This reward-enhancing effect of METH is believed to underlie its addictive properties [7–10]. The nucleus accumbens (NAc) is one of the key regions that mediate the rewarding effects of psychostimulants. A large number of cellular and molecular changes are observed in the NAc after repeated psychostimulant exposure. These changes include increased dopamine and glutamate release into the NAc [11, 12], subsequent increased dopamine receptor sensitivity [13], altered protein and non-coding RNA expression [14, 15] and changes in neuronal and synaptic plasticity of the NAc [16–18], thus emphasizing the importance of studying the underlying molecular mechanism of the effects of psychostimulants in detail.

As post-transcriptional regulators [19, 20], microRNAs (miRNAs) are expressed extensively in the brain and have various functions, being involved in processes from synaptic plasticity to neurological and psychological diseases [21–25]. In recent years, several miRNAs, such as miR-124, let-7d, miR-29c and miR-128, have been demonstrated to play roles in drug-induced reward [15, 26, 27]. Using miRNA sequencing (miRNA-Seq), we previously identified a small non-coding RNA, novel-m009C, which has not yet been annotated in miRbase. We characterized novel-m009C as a miRNA by annotating its precursor in the mouse genome (mm9) and generating its hairpin structure, and we found that its expression is significantly reduced in the NAc of METH-sensitized mice [28]. However, to date, information about whether novel-m009C is functionally implicated in METH reward is lacking.

In this study, we used mouse METH-induced CPP and hyperlocomotion models and viral infection to address this topic. Moreover, RNA immunoprecipitation-sequencing (RIP-Seq) and high-throughput RNA sequencing (RNA-Seq) followed by bioinformatics analysis and dual-luciferase reporter assays were employed to determine which targets and molecular pathways can be regulated. We also analyzed the human homolog of novel-m009C to preliminarily evaluate its application value.

## Material and methods

### Animals

The C57BL/6J mice (male, 7–8 weeks old) used in this study were purchased from Beijing Vital River Laboratory Animal Technology Co., Ltd. (Beijing, China). All mice were housed in cages in regulated environment (23 ± 1 °C, 50 ± 5% humidity) on a 12-h light/12-h dark cycle and were fed ad libitum. Mice were randomized by cage prior to drug treatment or before stereotaxic surgeries (i.e., each cage was assigned to each experimental condition or treatment: control or manipulation). The order of the mice was further randomized prior to behavioral tests.

All of the animal protocols used in this study were approved by the Institutional Animal Care and Use Committee of Xi’an Jiaotong University and were performed in accordance with the NIH Guide for Care and Use of Laboratory Animals. All efforts were made to minimize suffering and the number of animals used. All data derived from animal studies were analyzed by an experimenter blind to experimental conditions.

### Drug treatment

METH, cocaine and morphine were purchased from the National Institute for the Control of Pharmaceutical and Biological Products (Beijing, China) and were dissolved in 0.9% physiological saline. MK-801 (HY15084, MedChemExpress, China), SCH23390 (SCH) (D054, Sigma, USA), raclopride (RAC) (R121, Sigma, USA), spermidine trihydrochloride (SPD) (S2501, Sigma, USA) and arcaine sulfate salt (Arc) (D26320, Pfaltz & Bauer, USA) were also dissolved in 0.9% physiological saline. SKF-38393 (SKF) (GC17729, GLPBio, USA) and quinpirole (QNP) (Q102, Sigma, USA) were dissolved in double distilled water. Sucrose (Guangdong Guanghua Sci-Tec, China) was dissolved in water at a concentration of 1% (wt/vol).

For hyperlocomotion test, METH (2.0 mg/kg) [29], cocaine (10.0 mg/kg) [30], morphine (5.0 mg/kg) [31] and MK-801 (1.0 mg/kg) [32] were each administered via intraperitoneal (i.p.) injection, while SCH and RAC were intraperitoneally injected 30 min before METH injection at doses of 0.03 mg/kg and 2.0 mg/kg, respectively [33, 34]. For conditioned place preference (CPP) test, METH (1.0 mg/kg) [35], cocaine (10.0 mg/kg) [36] and morphine (10.0 mg/kg) [37] were each intraperitoneally injected, while SPD and Arc were intraperitoneally injected 30 min before METH at doses of 30.0 mg/kg and 3.0 mg/kg respectively [38]. SKF and QNP were each intraperitoneally injected daily at doses of 5.0 mg/kg and 0.5 mg/kg respectively for repeated 7 days [39, 40].

### Adeno-associated virus (AAV)

EGFP-expressing AAV_2/8_ for overexpressing novel-m009C precursor (AAV-m009C), a corresponding inhibitor-tough decoy RNA (TuD) (AAV-anti-m009C) and the corresponding control vectors (AAV-control and AAV-scrambled) were supplied by OBiO Technology, Shanghai, China (detailed information is provided in the Supplementary Information).

### Stereotaxic microinjection

Stereotaxic surgery and AAV microinjection were performed according to our previous publications [15, 27]. Twenty-eight days after surgery, AAV-microinjected mice were employed for behavioral tests. The coordinates for the NAc and the details of stereotaxic procedure are presented in the Supplementary Information.

### Behavioral tests

#### CPP test

The CPP apparatus (JLBeHv, China) was the same as that described in our previous study and the procedure was performed based on our previous study with modifications [35]. Details of the CPP test protocol are presented in the Supplementary Information.

#### Hyperlocomotion test

Here, we employed a locomotor sensitization regimen that was demonstrated to induce robust locomotion in our previous study [29]. Horizontal locomotor activity was assessed in metal test chambers (43 cm × 43 cm × 43 cm) and was quantified by a smart video tracking system (version 2.5; PanLab Technology for Bioresearch, Barcelona, Spain). Details of the hyperlocomotion test are presented in the Supplementary Information.

#### Sucrose preference test (SPT)

The SPT was performed after 1 week of habituation. On the two pre-test days, all mice were given two bottles of water or sucrose. Then, naïve mice were divided randomly into the water or sucrose group and were subjected to the SPT (sucrose group) or given water (water group) for 3 days. All AAV-injected mice were subjected to the SPT for one day to assess baseline sucrose preference before stereotaxic surgery. After stereotaxic injection, the mice were grouped according to the AAV they received. The mice were subjected to the SPT for 3 days (test 1-test 3) 28 days after the AAV microinjection. Details of the SPT protocol are presented in the Supplementary Information.

### Tissue collection

Twenty-four hours after the last injection, the mice were sacrificed, and the NAc was dissected out according to its structure and landmarks under a dissecting microscope [41]. The tissues were immediately frozen in liquid nitrogen until use.

### Reverse transcription

Total RNA was extracted using the miRNeasy Mini Kit (QIAGEN, USA). RNA concentration and quality were determined with a NanoDrop spectrophotometer (Thermo Scientific, USA). For miRNA detection, 500 ng of total RNA from each sample was reverse transcribed into cDNA using the Mir-X miRNA First-Strand Synthesis Kit (Takara, Japan). The reaction parameters were 37 °C for 60 min and 85 °C for 5 min. For mRNA detection, 500 ng of total RNA was reverse transcribed using PrimeScript RT Master Mix (TaKaRa, Japan) at the following parameters: 37 °C for 15 min, 85 °C for 5 s, and 4 °C for 5 min. All cDNA samples were stored at −20 °C for further use.

### Quantitative real-time PCR (qPCR)

qPCR was performed with SYBR Premix Ex Taq II (Takara, Japan) in a Bio-Rad iQ5 detection instrument (Bio-Rad, USA) under the following conditions: 95 °C for 30 s and 40 cycles of 95 °C for 5 s, 60 °C for 30 s and 72 °C for 30 s. U6 snRNA was used as an endogenous control for the measurement of novel-m009C expression, while *Gapdh* was used as a control for novel-m009C precursor and protein-coding gene expression measurements. Relative expression levels were determined using the 2^−ΔΔCt^ method [42]. The sequence of the primers are presented in the Supplementary Information.

### Western blot

The total protein was isolated with ice-cold RIPA buffer with protease inhibitors and the protein concentration in each sample was determined using a BCA protein assay kit (Pierce, Rockland, IL, USA). Primary antibody against Grin1(rabbit anti-NMDAR1, ab109182, Abcam) was used at 1:1000 dilutions and goat anti-rabbit lgG horseradish peroxidase-conjugated secondary antibody (Proteintech, USA) was used at 1:2000 dilution. Details of the western blot protocol are presented in the Supplementary Information.

### RIP-Seq and RNA-Seq

A total of 24 mice were used for RIP-Seq and RNA-Seq analyses. We used NAc samples extracted from mice in the AAV-m009C group (*n* = 12) and AAV-control group of mice (*n* = 12) that had been subjected to METH-induced hyperlocomotion test. We pooled the lysates of four injected NAc samples from each group to produce a single sample, and three replicate samples from each group were prepared for both RIP-Seq and RNA-Seq.Thus, the same pooled samples were used for RIP-Seq and RNA-Seq. The experimental details are presented in the Supplementary Information.

### Bioinformatic analyses

Target site predictions were performed by RNAhybrid (v2.1.2)+svm_light (v6.01) and Miranda (v3.3a). IPA software (version: 2018 summer) (Ingenuity Systems, Redwood City, CA, USA; apps.ingenuity.com) was used to annotate the molecular functions and regulatory mechanisms of the potential targets of novel-m009C. MEGAX software (version 10.1.8, USA) was used for the phylogenetic analysis. Details of these analyses are presented in the Supplementary Information.

### Fluorescence in situ hybridization (FISH)

Paraffin sections of brains from METH-sensitized and saline-control mice (*n* = 3/group) were used for FISH. Specific riboprobe for novel-m009C was fluorescently labeled (5’CY3 and 3’CY3, GenePharma, Shanghai) and used at a final labeled nucleotide concentration of 1.3 μM/l. Riboprobe for negative control was scrambled sequence labeled with 5’CY3 and 3’CY3. Detailed protocol was presented in Supplementary Information. Note that the FISH procedure of the brain sections, the image acquisition and the analyses of the images were performed blindly without knowledge of the treatment.

### Dual-luciferase reporter assay

A pMIR reporter luciferase miRNA target expression vector (OBIO Tech. Shanghai, PR China) and a pRL-CMV Renilla luciferase vector (Promega, USA) were used for dual-luciferase reporter assays. HEK293T cells were obtained from the Cell Bank of the Chinese Academy of Sciences, which were authenticated and tested for mycoplasma contamination. Luciferase activity was quantified by a luminometer (Spark 10 M, Tecan, Switzerland). The experimental details are presented in the Supplementary Information.

### Statistical analysis

Statistical analyses were performed using SPSS (version 20.0) and GraphPad Prism 7.0a. For the hyperlocomotion test and SPT data, one-way or mixed-design repeated measures ANOVA was carried out to verify the significance of main or interaction effects, with day as the within-subject variable and treatment as the between-subject factor. For the CPP test, the data were analyzed by two-way ANOVA, with test as the within-subject variable and treatment as the between-subject factor. qPCR data was analyzed by independent *t*-test with two-tailed analysis or two-way ANOVA. Western blot data and FISH data were analyzed by independent *t*-test with two-tailed analysis. Sample sizes (*n*) are indicated in the figure legends. No statistical methods were used to determine the sample sizes, but the number of experimental subjects is similar to sample sizes routinely used in our laboratory and in the field for similar experiments. All data were normally distributed and variance was similar between groups. All values were expressed as the mean ± standard error of the mean (SEM), and *P* < 0.05 was considered statistically significant.

## Results

### Novel-m009C expression in the NAc was significantly decreased by METH

**Fig. 1:**
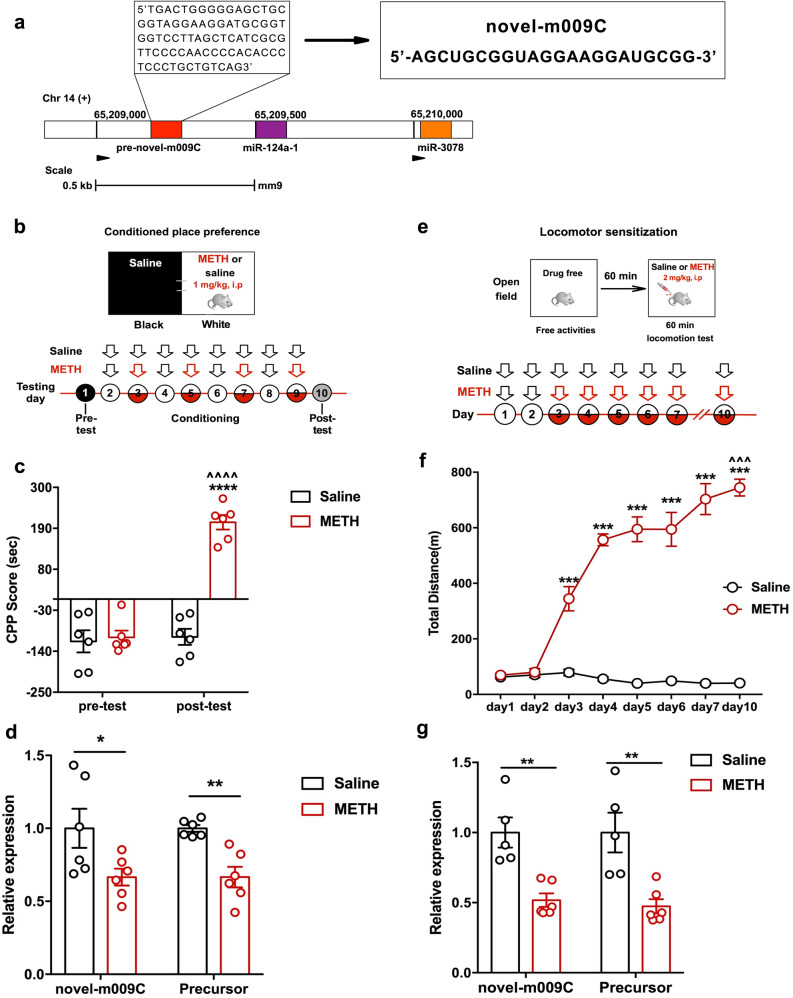
Novel-m009C is a miRNA of mice and its expression was substantially reduced by METH. **a** The sequence of novel-m009C and its precursor in mice genome. **b** METH-induced CPP protocol. **c** METH-induced CPP in mice. Two-way ANOVA. METH: *F*_(1, 20)_ = 49.36, *P* < 0.0001; test: *F*_(1, 20)_ = 50.14, *P* < 0.0001; METH × test: *F*_(1, 20)_ = 42.87, *P* < 0.0001. ****P < 0.0001, different from the saline group in the post-test; ^^^^*P* < 0.0001, different from the pre-test score of the same group; *n* = 6. **d** Significant decrease in the expression of novel-m009C and its precursor in the NAc of mice subjected to the METH-induced CPP test. NAc samples for qPCR were collected after the post-test. Independent *t*-test. Novel-m009C fold change: *t*_(10)_ = 2.292; precursor fold change: *t*_(10)_ = 4.516; *P < 0.05, **P < 0.01; *n* = 6. **e** Protocol of the METH-induced locomotor sensitization test. **f** METH-induced locomotor sensitization of mice. One-way repeated measures ANOVA. METH: *F*_(1,10)_ = 173.114, *P* < 0.0001; day: *F*_(7, 70)_ = 88.122, *P* < 0.0001; day × METH: *F*_(7, 70)_ = 105.414, *P* < 0.0001. ***P < 0.001, different from the saline group on each day; ^^^*P* < 0.001, different from the same group on day 3; *n* = 6. **g** Substantial reduction in the expression of novel-m009C and its precursor in the NAc of mice after METH sensitization. NAc samples for qPCR were collected 24 h after the last injection. Independent *t*-test. novel-m009C fold change: *t*_(9)_ = 4.363; precursor: *t*_(9)_ = 3.77; **P < 0.01; *n* = 5–6. All values are presented as the mean ± SEM.

Novel-m009C is a novel miRNA that we identified previously (Fig. 1a). The precursor sequence of novel-m009C (pre-novel-m009C), which was validated through genomic annotation and hairpin structure generation, is located on chromosome 14 of the mouse genome and is close to the *miR-124a-1/miR-3078* cluster (<1 kb, Fig. 1a) [28]. Novel-m009C is the mature miRNA that is spliced from the 5’arm of the precursor hairpin.

To verify the role of novel-m009C in METH abuse, we firstly performed qPCR to examine the expression changes of both novel-m009C and its precursor, as well as its closest miRNA cluster-miR-124a-1/miR-3078, in the NAc of mice using METH-induced CPP and hyperlocomotion models. We found that in mice subjected to the METH-induced CPP test and hyperlocomotion test, the expression of both novel-m009C and its precursor were substantially decreased in the NAc (*P* < 0.05) (Fig. 1b–g), which were similar to the change of miR-124-3p but not miR-3078 in the NAc of mice in response to METH (Supplemental Fig. 1). Secondly, we examined the expression data of novel-m009C in the NAc, as well as caudate putamen (CPu) and anterior cingulate cortex (ACC), in METH versus saline-treated mice by FISH assay. Although novel-m009C was found to be ubiquitously distributed in the NAc, CPu and ACC, it was significantly downregulated in the NAc of METH-treated mice, while there were not any expression changes in the CPu or ACC (Supplementary Fig. 2). The downregulated expression of novel-m009C in the NAc quantified by FISH (Supplemental Fig. 2) was in accordance with the data from qPCR (Fig. 1d, g). These findings indicated that novel-m009C was not specific to the NAc, but was significantly changed in the NAc when mice were exposed to METH. Moreover, significant reduction in novel-m009C expression was also observed in the NAc of mice subjected to cocaine, but was not captured in the NAc of mice subjected to an opioid drug-morphine or a psychiatric stimuli-MK-801 (Supplemental Fig. 3). In sum, the abovementioned findings suggested that a reduction of novel-m009C expression in the NAc may be involved in the hyper-rewarding effects of METH and cocaine.”

### METH may reduce novel-m009C expression by activating dopamine receptor D1 (D1R) and dopamine receptor D2 (D2R) in the NAc

**Fig. 2:**
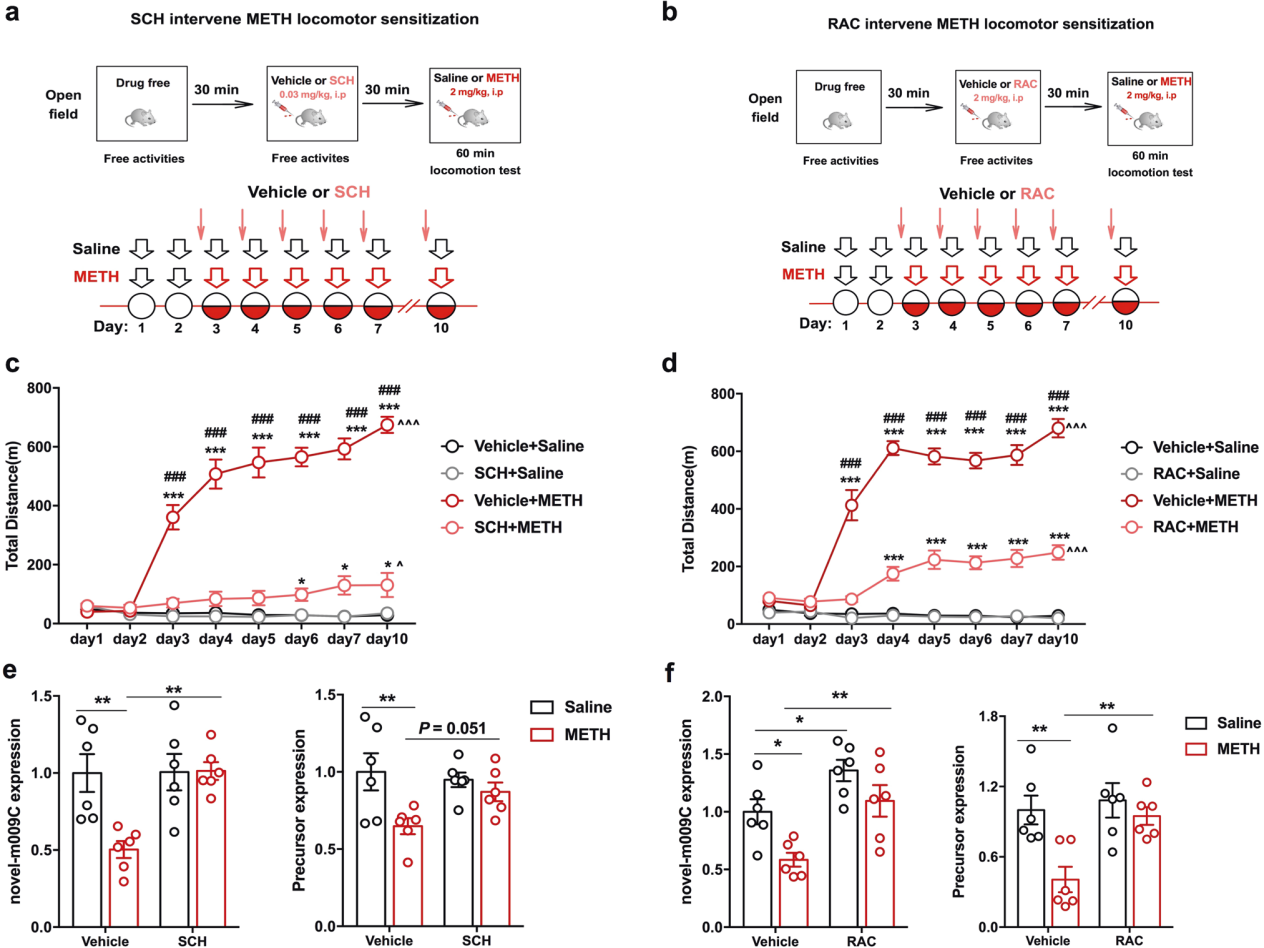
The METH-induced decrease in novel-m009C expression in the NAc of mice was reversed by a D1R or D2R antagonist. Timeline of SCH (**a**) or RAC (**b**) pre-treatment prior to the METH-induced locomotor sensitization test. SCH, RAC or vehicle was given 30 min prior to saline or METH injection. NAc samples for qPCR were collected 24 h after the last injection. SCH (**c**) or RAC (**d**) pre-treatment significantly attenuated the hyperlocomotion induced by METH. Mixed-design repeated measures ANOVA with a post hoc multiple comparison test. SCH pre-treatment experiment: METH: *F*_(1, 24)_ = 178.697, *P* < 0.001; SCH: *F*_(1, 24)_ = 100.039, *P* < 0.001; day: *F*_(7, 168)_ = 42.688, *P* < 0.001; day × METH: *F*_(7, 168)_ = 52.158, *P* < 0.001; day × SCH: *F*_(7, 168)_ = 31.872, *P* < 0.001; METH × SCH: *F*_(1,24)_ = 97.336, *P* < 0.001; day × SCH × METH: *F*_(1, 24)_ = 97.336, *P* < 0.001. RAC pre-treatment: METH: *F*_(1, 24)_ = 310.569, *P* < 0.001; RAC: *F*_(1, 24)_ = 82.320, *P* < 0.001; day: *F*_(7, 168)_ = 82.759, *P* < 0.001; day × METH: *F*_(7, 168)_ = 98.008, *P* < 0.001; METH × RAC: *F*_(1, 24)_ = 76.882, *P* < 0.001; day × RAC: *F*_(7, 168)_ = 32.951, *P* < 0.001; day × RAC × METH: *F*_(1, 24)_ = 31.666, *P* < 0.001. *P < 0.05, ***P < 0.001, different from the paired saline group mice on each injection day; ^*P* < 0.05, ^^^*P* < 0.001, different from the same group on day 3; ^###^*P* < 0.001, different from the vehicle + METH group; *n* = 6–8. SCH (**e**) or RAC (**f**) inhibited the METH-induced decrease in the expression of novel-m009C and its precursor in the NAc of mice. Two-way ANOVA followed by a post hoc multiple comparison test. SCH pre-treatment experiment: novel-m009C fold change: METH: *F*_(1, 20)_ = 6.778, *P* = 0.017; SCH: *F*_(1, 20)_ = 7.529, *P* = 0.0125; METH × SCH: *F*_(1,20)_ = 7.201, *P* = 0.0143; **P < 0.01; precursor fold change: METH: *F*_(1, 20)_ = 8.029, *P* = 0.0103; SCH: *F*_(1, 20)_ = 1.272, *P* = 0.2728; METH × SCH: *F*_(1,20)_ = 3.268, *P* = 0.0857. **P < 0.01; *n* = 6. RAC pre-treatment experiment: novel-m009C fold change: METH: *F*_(1, 20)_ = 10.84, *P* = 0.0036; RAC: *F*_(1, 20)_ = 17.71, *P* = 0.0004; METH × SCH: *F*_(1, 20)_ = 0.5452, *P* = 0.4689; *P < 0.05, **P < 0.01; precursor fold change: METH: *F*_(1, 20)_ = 9.739, *P* = 0.0054; RAC: *F*_(1, 20)_ = 7.17, *P* = 0.0145; METH × RAC: *F*_(1, 20)_ = 3.882, *P* = 0.0628. **P < 0.01; *n* = 6. All values are presented as the mean ± SEM.

Considering that D1R and D2R are both essential for the rewarding effects of psychoactive drugs [16, 43], we firstly applied SCH (a D1R antagonist) and RAC (a D2R antagonist) to determine whether inhibition of D1R or D2R activity could modulate novel-m009C expression in the NAc of mice upon METH treatment (Fig. 2a, b). SCH and RAC suppressed the METH-induced locomotor sensitization of mice (*P* < 0.001) (Fig. 2c, d). Pre-treatment with either SCH or RAC reversed the METH-induced downregulation of the expression of novel-m009C and its precursor (*P* < 0.05, Fig. 2e, f). Secondly, we examined the influence of D1R agonist (SKF) and D2R agonist (QNP) on the expression of novel-m009C in the NAc of mice. The results indicated that repeated treatments of SKF and QNP both significantly reduced the expression of novel-m009C and its precursor in the NAc of mice (Supplemental Fig. 4). These results indicated that METH may reduce novel-m009C expression through activating D1R and D2R signaling in the NAc.

### Alteration in novel-m009C expression in the NAc regulated METH reward

#### Overexpression of novel-m009C in the NAc decreased the rewarding effect of METH

**Fig. 3:**
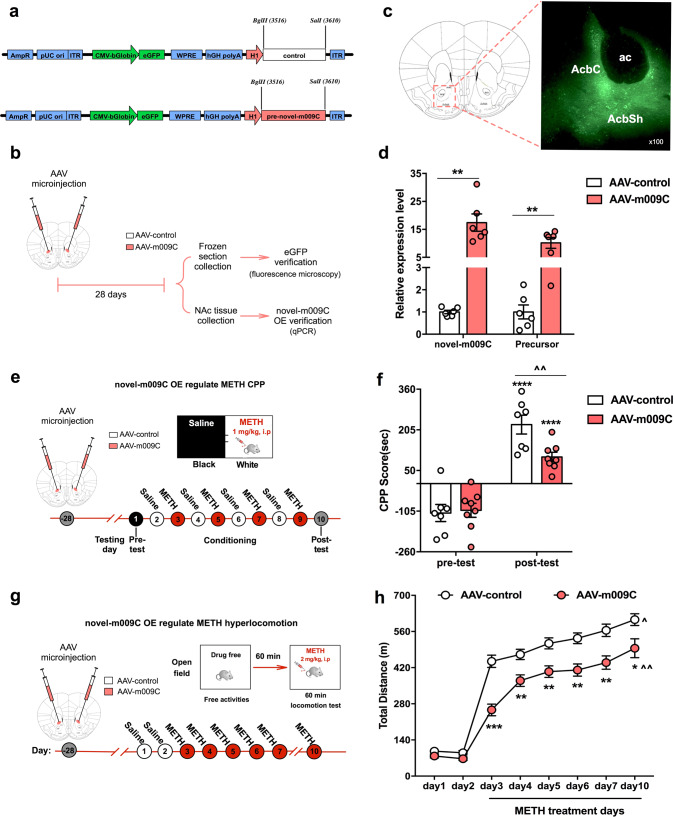
Overexpression of novel-m009C in the NAc attenuated METH-induced CPP and hyperlocomotion in mice. **a** Schematic of the control (AAV-control) and novel-m009C precursor expression AAV vectors. **b** Timeline of the viral infection experiments performed to validate the location of microinfusion and overexpression efficacy through evaluation of the expression of eGFP and novel-m009C. **c** eGFP was locally expressed in the NAc of mice, as visualized by fluorescence microscopy. **d** The expression of novel-m009C and its precursor was substantially heightened in the NAc of AAV-m009C-injected mice. Independent *t*-test. Novel-m009C fold change: *t*_(10)_ = 5.313; precursor fold change: *t*_(10)_ = 4.695; **P < 0.01; *n* = 6. **e** Timeline of AAV injection and CPP test. **f** The regulatory effect of AAV-m009C in the NAc decreased METH-induced CPP. Two-way ANOVA. Virus: *F*_(1, 26)_ = 3.936, *P* = 0.0579; test: *F*_(1, 26)_ = 90.13, *P* < 0.0001; virus × test: *F*_(1, 26)_ = 5.54, *P* = 0.0264; ^^*P* < 0.01, different from the AAV-control group in the post-test; ****P < 0.0001, different from the pre-test score of the same group; *n* = 7–8. **g** Timeline of AAV injection and METH-induced locomotor sensitization test. **h** The regulatory effect of AAV-m009C in the NAc decreased METH-induced hyperlocomotion. One-way repeated measures ANOVA. Virus: *F*_(1, 22)_ = 22.595, *P* < 0.001; day: *F*_(7, 154)_ = 258.163, *P* < 0.001; virus × day: *F*_(7, 154)_ = 5.925, *P* < 0.001; *P < 0.05, **P < 0.01, ***P < 0.001, different from the AAV-control group; ^*P* < 0.05, ^^*P* < 0.01, different from the same group on day 3; *n* = 12. All values are presented as the mean ± SEM.

Next, we assessed the effect of overexpressing novel-m009C in the NAc on METH-induced CPP and METH hyperlocomotion. We used an AAV expressing the precursor of novel-m009C (AAV-m009C) to overexpress novel-m009C and empty vector (AAV-control) as a control (Fig. 3a). AAV-mediated eGFP expression was restricted to the NAc (Fig. 3b, c). The expression of both novel-m009C and its precursor was significantly increased more than 10-fold in the NAc of AAV-m009C-injected mice (Fig. 3d), demonstrating that AAV-m009C induced significant overexpression of novel-m009C. In the CPP test (Fig. 3e), the mice in the AAV-m009C- and AAV-control-injected groups displayed similar CPP scores in the CPP pre-test (Fig. 3f). Although all mice exhibited METH-induced CPP, AAV-m009C-injected mice showed a significantly decreased METH-induced CPP score compared with that of AAV-control mice (Fig. 3f). In the hyperlocomotion test (Fig. 3g), overexpression of novel-m009C did not alter the locomotor abilities of mice, as the total distance traveled did not differ between AAV-m009C- and AAV-control-injected mice on days 1–2 (Fig. 3h). When exposed to METH, AAV-m009C-injected mice expressed significantly attenuated locomotor responses to METH on each day of drug treatments (Fig. 3h).

#### Inhibition of novel-m009C in the NAc increased the rewarding effect of METH

**Fig. 4:**
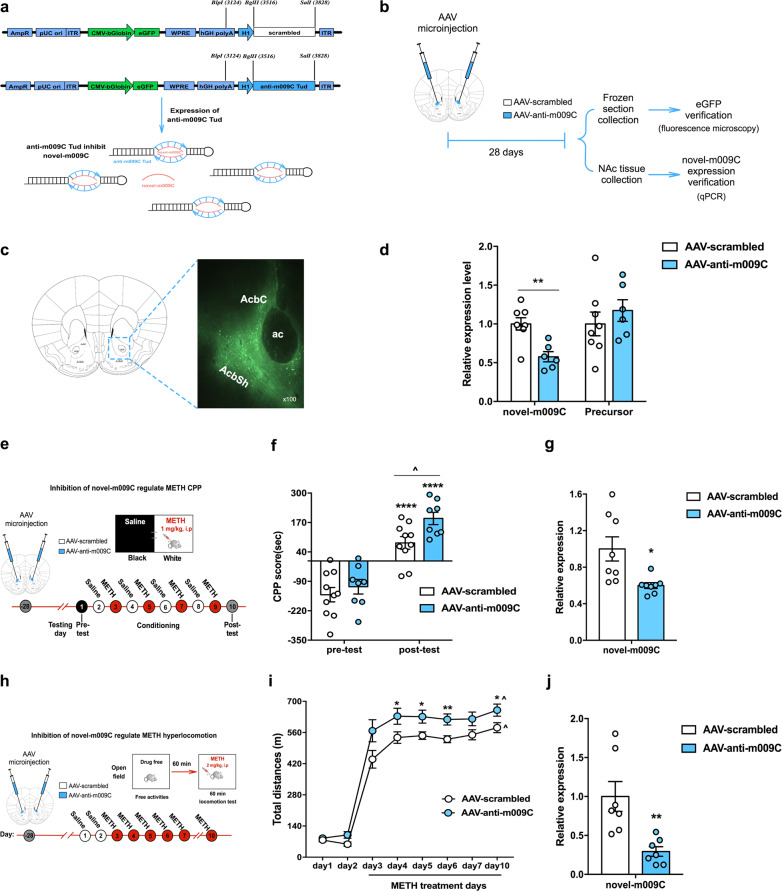
Inhibition of novel-m009C in the NAc enhanced METH-induced CPP and hyperlocomotion in mice. **a** Schematic of the scrambled control (AAV-scrambled) and anti-m009C TuD RNA expression AAV vectors. Anti-m009C TuD RNA was used to specifically inhibit novel-m009C. **b** Timeline of the viral infection experiments performed to validate the location of microinfusion and the inhibition efficacy through evaluation of the expression of eGFP and novel-m009C. **c** eGFP showed local expression in the NAc of mice, as visualized by fluorescence microscopy. **d** The expression of novel-m009C but not its precursor significantly decreased in the NAc of AAV-anti-m009C-injected mice. Independent *t*-test. Novel-m009C fold change: *t*_(12)_ = 3.896; precursor: *t*_(12)_ = 0.8054; **P < 0.01; *n* = 6–8. **e** Timeline of AAV injection and the CPP test. **f** The regulatory effect of AAV-anti-m009C in the NAc increased METH-induced CPP. Two-way ANOVA. Virus: *F*_(1, 32)_ = 5.643, *P* < 0.05; test: *F*_(1, 32)_ = 76.73, *P* < 0.0001; virus × test: *F*_(1, 32)_ = 1.46, *P* = 0.2357. ****P < 0.0001, different from the pre-test score of the same group; ^*P* < 0.05, different from the AAV-scrambled group; *n* = 8–10. **g** Novel-m009C was significantly decreased in the NAc of AAV-anti-m009C-injected mice following METH-CPP test. Independent *t*-test. Novel-m009C fold change: *t*_(14)_ = 2.949; *P < 0.05; *n* = 8. **h** Timeline of AAV injection and the METH-induced locomotor sensitization test. **i** The regulatory effect of AAV-anti-m009C in the NAc increased METH-induced hyperlocomotion. One-way repeated measures ANOVA. Virus: *F*_(1, 20)_ = 7.861, *P* < 0.05; day: *F*_(7, 14)_ = 304.456, *P* < 0.001; virus × day: *F*_(7, 140)_ = 1.848, *P* = 0.155. *P < 0.05, **P < 0.01, different from the AAV-scrambled group; ^*P* < 0.01, different from the same group on day 3; *n* = 9–13. **j** Novel-m009C was significantly decreased in the NAc of AAV-anti-m009C-injected mice following METH-hyperlocomotion test. Independent *t*-test. Novel-m009C fold change: *t*_(12)_ = 3.507; **P < 0.01; *n* = 7. All values are presented as the mean ± SEM.

We then evaluated whether inhibiting novel-m009C expression in the NAc enhanced METH-induced CPP and METH-induced hyperlocomotion. We used an AAV vector expressing the specific inhibitor anti-m009C TuD (AAV-anti-m009C) to inhibit novel-m009C expression with high specificity in vivo (Fig. 4a). An AAV expressing a scrambled sequence (AAV-scrambled) was used as a control (Fig. 4a). We observed that AAV-mediated expression of eGFP was restricted to the NAc (Fig. 4b, c). The expression of novel-m009C but not its precursor was decreased notably in the NAc of AAV-anti-m009C-injected mice (*P* < 0.05) (Fig. 4d), indicating the inhibitory effect of AAV-anti-m009C. In the CPP test (Fig. 4e), AAV microinjection did not change the preference of mice, as both groups of mice exhibited similar CPP scores in the pre-test (Fig. 4f). After METH conditioning, AAV-scrambled-injected mice exhibited a normal preference for the METH-paired compartment. However, this preference for the METH-paired compartment was enhanced in AAV-anti-m009C-injected mice (*P* < 0.05) (Fig. 4f). In the hyperlocomotion test (Fig. 4h), AAV-anti-m009C-injected mice exhibited a level of locomotor activity that was indistinguishable from that of AAV-scrambled-injected mice on day 1 and day 2, also demonstrating that inhibition of novel-m009C expression did not alter the basal locomotor activity of mice. When METH was given, AAV-anti-m009C-injected mice displayed an enhanced locomotor response to METH (days 4–6 and day 10) (*P* < 0.05) (Fig. 4i). Significantly decreased expression levels of novel-m009C in the NAc following METH-CPP and hyperlocomotion test were examined respectively, and the results confirmed that AAV-anti-m009C significantly decreased novel-m009C expression on the basis of METH treatment (Fig. 4g, j).

#### Alteration of the levels of novel-m009C in the NAc did not affect the natural preference of mice for sucrose

We further examined whether modification of the levels of novel-m009C in the NAc affects the preference of mice for sucrose, a mild natural reward. We found that the expression of neither novel-m009C nor its precursor showed significant changes in response to sucrose (Supplemental Fig. 5a–c). Importantly, natural preference for sucrose was not affected in either mice overexpressing novel-m009C or mice in which novel-m009C expression was inhibited compared to corresponding control mice (Supplemental Fig. 5d–h). Collectively, these findings demonstrated that novel-m009C in the NAc is required for METH’s hyper-rewarding effect but not for a broader reward response, indicating an important role for novel-m009C in the development of METH addiction.

### Target genes that may be involved in novel-m009C-mediated regulation of METH reward

#### Novel-m009C- targeted genes involved in neuronal morphology and synaptic plasticity

**Fig. 5:**
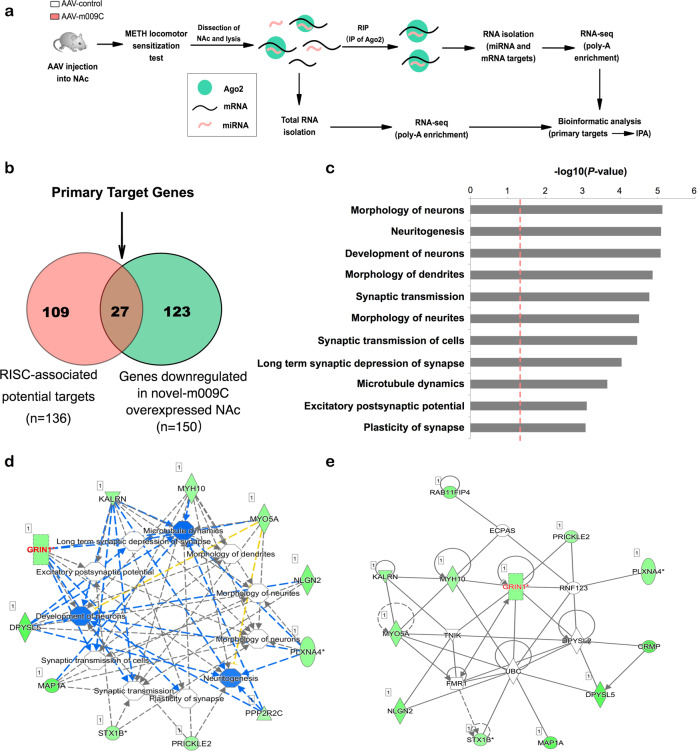
Novel-m009C regulates potential target genes involved in neuronal morphology and synaptic activities. **a** Procedure used for RIP-Seq and RNA-Seq. The mice were microinjected with AAV-m009C and AAV-control and subjected to the METH-induced locomotor sensitization test. Twenty-four hours after the final injection, NAc tissue was collected and lysed. Half of each NAc homogenate was used for immunoprecipitation (IP) of Ago2, and RNA that cross-linked with Ago2 was then isolated for high-throughput sequencing. RNA extracted from the other half of each homogenate was used for high-throughput sequencing; *n* = 3 pooled homogenates per group (*see Methods*). Sequencing data were subjected to bioinformatics analysis to identify the primary targets of novel-m009C, and IPA was performed to analyze the functional networks of the targets. **b** Venn diagram showing the RISC-associated mRNA targets of novel-m009C (red) and mRNAs with expression levels that were downregulated in the NAc of AAV-m009C-injected mice (green). The 27 overlapping genes were considered direct novel-m009C targets. **c** The -log10 (*P* value) of the disease and functional terms of the 27 target genes identified by IPA are shown. The red dotted line indicates *P* = 0.05. **d** Interaction network of the 27 direct targets showing the functions and the target genes involved. Correlated connections and networks between the target genes and functional terms were analyzed according to the IPA database. The shape of each gene indicates its functional class, and genes that are green are those with downregulated expression. Functional terms in blue indicate an inhibitory state. The relationships between genes and functional terms are indicated by dotted lines of different colors, which indicate the degree of interaction (blue: inhibition, yellow: inconsistent with the state of the downstream molecule, gray: effect not predicted). **e** The gene network of these primary target genes. Genes with expression levels that were directly downregulated by AAV-m009C are indicated in green. The high abundance of Grin1 (encoding NMDAR subunit 1) among the targets in red underscores the potential of novel-m009C to control signaling processes associated with neuronal morphology and synaptic activities.

To identify targets of novel-m009C that are responsible for its effects on METH reward, we used RIP-Seq to analyze mRNAs associated with Ago2 in the NAc of AAV-m009C- and AAV-control-injected mice subjected to the METH-induced hyperlocomotion test as a pool of mRNAs in the NAc that may be suppressed by novel-m009C (Fig. 5a). Moreover, we employed RNA-Seq to analyze the mRNA expression profiles of the same samples from AAV-m009C- and AAV-control-injected mice after METH treatment (Fig. 5a). The primary targets of novel-m009C in the regulation of METH exposure might be predicted as targets and be both enriched by Ago2 and display downregulation mRNA levels. Through this strategy, 27 genes were found to be RISC-associated novel-m009C target genes (Fig. 5b and Supplemental table 1). According to IPA, these 27 genes were significantly enriched in processes involved in neuronal morphology and synaptic plasticity (Fig. 5c and Supplemental table 2). These results indicated the ability of novel-m009C to affect molecular processes that are intrinsically linked to the regulation of METH-induced synaptic transmission. The high abundance of Grin1 (NMDAR subunit 1) among the targets also underscored the potential ability of novel-m009C to control signaling processes associated with synaptic transmission (Fig. 5d, e).

#### Grin1 may be involved in novel-m009C regulating METH reward

**Fig. 6:**
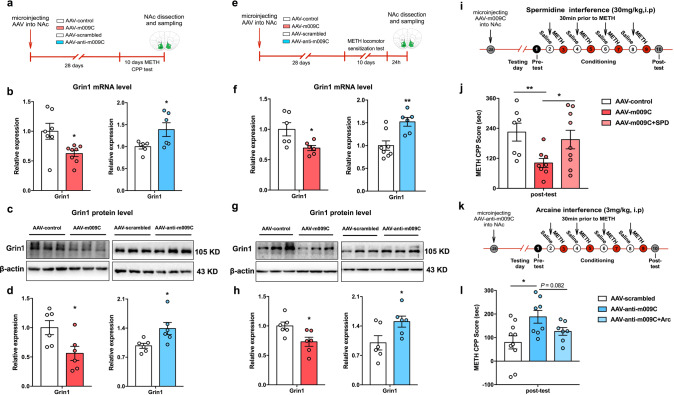
Grin1 may be a target of novel-m009C in the regulation of METH-induced CPP and hyperlocomotion. **a** Procedure for measuring Grin1 expression in the NAc of AAV-injected mice following the METH-induced CPP test. NAc samples were collected after the post-test. **b** The mRNA level of Grin1 expression was downregulated and upregulated in the NAc of mice in which novel-m009C was overexpression and inhibited, respectively, following the CPP test. Independent *t*-test. Overexpression: *t*_(13)_ = 2.745; inhibition: *t*_(10)_ = 2.35; *P < 0.05, different from the corresponding control group; *n* = 6–8. Representative western blots (**c**) and graphs (**d**) showing the protein level of Grin1 expression was downregulated and upregulated in the NAc of mice in which novel-m009C was overexpressed and inhibited, respectively, following the CPP test. Independent *t*-test. Overexpression: *t*_(10)_ = 2.583; inhibition: *t*_(10)_ = 2.639; *P < 0.05, different from the corresponding control group; *n* = 6. **e** Procedure for measuring Grin1 expression in the NAc of AAV-injected mice following the METH-induced hyperlocomotion test. NAc samples were collected 24 h after the final METH injection. **f** The mRNA level of Grin1 expression was downregulated and upregulated in the NAc of mice in which novel-m009C was overexpression and inhibited, respectively, following the METH-induced hyperlocomotion test. Independent *t*-test. Overexpression: *t*_(10)_ = 2.646; inhibition: *t*_(12)_ = 3.567. *P < 0.05, **P < 0.01, different from the corresponding control group; *n* = 6–8. Representative western blots (**g**) and graphs (**h**) showing the protein level of Grin1 expression was downregulated and upregulated in the NAc of mice in which novel-m009C was overexpressed and inhibited, respectively, following the hyperlocomotion test. Independent *t*-test. Overexpression: *t*_(10)_ = 2.667; inhibition: *t*_(10)_ = 2.35; *P < 0.05, different from the corresponding control group; *n* = 6. **i** Procedure for intervening in the AAV-m009C-mediated regulation of METH-induced CPP with SPD. SPD (30 mg/kg, i.p.) was given 30 min before METH injection in the conditioning phase. **j** SPD treatment reversed the decreasing effect of AAV-m009C on METH-induced CPP. The CPP score of AAV-m009C + SPD-treated mice was compared with that of AAV-m009C- and AAV-control-treated mice. Independent *t*-test: *t*_1 (13)_ = 3.192, *t*_2 (11.5)_ = 2.265, *t*_3 (14)_ = 0.5665; *P < 0.05, **P < 0.01; *n* = 7–9. **k** Procedure for intervening in the AAV-anti-m009C-mediation regulation of METH-induced CPP with Arc. Arc (3.0 mg/kg, i.p.) was given 30 min before METH injection in the conditioning phase. **l** The effect of AAV-anti-m009C on METH-induced CPP showed an attenuated trend following Arc intervention. The CPP score of AAV-anti-m009C +Arc-treated mice was compared with that of AAV-anti-m009C- and AAV-scrambled-treated mice. Independent *t*-test: *t*_1 (16)_ = 2.742, *t*_2 (13)_ = 1.884, *t*_3 (15)_ = 1.28; *P < 0.05; *n* = 7–10. All values are presented as the mean ± SEM.

Thus, we determined whether novel-m009C regulates METH reward through targeting Grin1. Firstly, we found that Grin1 expression, both in mRNA level and protein level, was negatively regulated by novel-m009C following METH-induced CPP and hyperlocomotion tests (Fig. 6a–h), but was not changed in the NAc of either novel-m009C-overexpressing mice or mice in which novel-m009C expression was inhibited following the SPT (Supplemental Fig. 6a–c). Secondly, a dual-luciferase reporter assay indicated that the Luc/R-Luc ratio was significantly decreased when novel-m009C was co-transfected with the reporter vector containing the 3’UTR of Grin1 and the ratio was a small increase (+5%) when novel-m009C was co-transfection with the reporter vector containing the Grin1 3’-UTR (Mut) in which one target site was mutated (Supplemental Fig. 6d, e). This effect of the mutant Grin1 3’UTR may result from the atypical binding site directly targeting the 3’UTR of Grin1 in the cells [44]. Grin1 encodes NMDAR subunit 1, whose expression changes reflect an alteration in NMDARs. We then used SPD, an NMDAR agonist, and Arc, an NMDAR antagonist, to intervene with the regulation of METH-induced CPP by AAV-m009C and AAV-anti-m009C. We found that AAV-m009C-mediated attenuation of METH-induced CPP was blunted when SPD was given (Fig. 6i, j) and that accordingly, AAV-anti-m009C-mediated enhancement of METH-induced CPP was showed an attenuated trend when Arc was injected (Fig. 6k, l). We finally verified the expression of Grin1 in the NAc of mice following METH exposure. It was shown that both mRNA and protein levels of Grin1 were significantly decreased in the NAc of mice in response to METH (Supplemental Fig. 6f–i). Collectively, these findings suggested that Grin1 may be involved in novel-m009C regulating METH reward.

### Novel-m009C may be homologous to hsa-miR-604

Novel-m009C is a miRNA that is found in mouse genome, and increasing its expression level in the NAc can decrease METH reward; thus, identifying its human homologs is essential for further application. Mature miRNAs are highly conserved across different species and the “seed” sequence (2–8 nt from the mature miRNA 5’end), which is a conserved heptametrical sequence, is the functional execution region of a miRNA, that the high functional cost of even single nucleotide changes within seed regions is consistent with their high sequence conservation among miRNA families both within and between species [45–49]. Thus, we blasted the “seed” sequence of novel-m009C with all miRNAs that were annotated in miRBase (version 22.0), and found ten miRNAs from other species contained the same “seed” sequence of novel-m009C (Supplemental Fig. 7a). Moreover, among these ten miRNAs, the highest similarity was shown between the whole sequence of novel-m009C and miR-604 by sequence blasting (Supplemental Fig. 7a). MiR-604 is a primate-specific miRNA that has only been found to be expressed in primates (hsa-miR-604, ppy-miR-604, ptr-miR-604, and mml-miR-604). Therefore, we analyzed the phylogenetic tree between novel-m009C and primate-derived miR-604, and found that hsa-miR-604 and novel-m009C are the closest in evolutionary relationship (Supplemental Fig. 7b). The abovementioned findings suggested that hsa-miR-604 may be the human homologs of novel-m009C.

We performed a dual-luciferase reporter assay again to confirm whether hsa-miR-604 can target Grin1 in *Homo sapiens*. We predicted two target sites of hsa-miR-604 on Grin1 (Supplemental Fig. 7c). The results showed that the Luc/R-Luc ratio was significantly decreased when hsa-miR-604 was co-transfected with the Grin1 3’UTR (WT) but was significantly increased (+17%) when target sites within the Grin1 3’-UTR were mutated (Supplemental Fig. 7d). These results suggested that hsa-miR-604 may exert a similar biological function as novel-m009C by targeting Grin1.

## Discussion

In this study, we found that the substantial reduction in novel-m009C expression in the NAc of mice was resulted from psychostimulants (METH and cocaine) treatment, neither morphine, MK-801 nor sucrose induced any expression changes in novel-m009C. This reduction in expression could be regulated by both D1R and D2R. METH is a widely abused psychostimulant and has been demonstrated to produce a strong rewarding effect by inducing hyperdopaminergia in the NAc, which is believed to underlie its addictive effects [6, 10]. Activation of D1R and D2R has been implicated in the drug-induced reinforcement behaviors [16, 43, 50, 51]. There are evidences showing that D2R activation is essential for D1R-mediated effects in response to psychostimulants [52], and the ERK/MAPK intracellular signaling pathway, which plays critical roles in the long-term behavioral effects of drug abuse [53, 54], is required for both D1R- and D2R-mediated locomotor phenotype [55]. In addition, a D1-D2 receptor heteromer pathway has been shown to lead to the activation of calcium signal cascade following increases in extracellular dopamine levels [56]. Based on these evidences, our results suggested that METH-reduced the expression of novel-m009C in the NAc may possibly through activation of both D1R and D2R or the D1R-D2R heteromer pathway, and that the ERK/MAPK signaling pathway and the calcium signal cascade may be involved in regulating this reduction of novel-m009C. These preliminary results in our study are hypothesis generating and of interest for future studies, which would be desirable for further confirmation.

By suppressing mRNA expression or translation, miRNAs act as post-transcriptional regulators in various cellular processes. Thus, considering the bidirectional impact of novel-m009C manipulation on METH reward, the targets and molecular pathways that are most likely regulated should be identified. Here, by using RIP-Seq and RNA-Seq, we found 27 genes involved in modulating changes in neuronal morphology and synaptic plasticity may be the targets of novel-m009C. The fact that only ~20% of the potential RISC-associated novel-m009C targets display decreased expression is likely to reflect the known redundancy among miRNAs. Many mRNAs are regulated by more than one miRNA [44, 57], thus limiting the actual impact of individual miRNA over-expression on the expression of miRNA targets in vivo. Among these potential targets, we verified that Grin1 may be involved in novel-m009C regulating METH rewards. A previous study demonstrated that deleting Grin1 in NAc D1R-expressing medium spiny neurons (MSNs) significantly attenuates amphetamine sensitization [58]. Here, we found that activation of D1R whether by psychostimulants (METH and cocaine) or its specific agonist significantly reduced the expression level of novel-m009C in the NAc, while inhibition of D1R significantly attenuated METH sensitization and inhibited the METH-reduced levels of novel-m009C in the NAc. Overexpressing NAc novel-m009C attenuated the rewarding effects of METH and significantly decreased Grin1 expression. Activated NMDAR could reverse the attenuating effect of novel-m009C overexpression on METH reward. Combining these findings together, we hypothesized that METH exposure may activate D1R, through which decreased novel-m009C levels in the NAc, thus increasing Grin1 expression and ultimately affecting NMDAR activity and exerting rewarding effects. It was reported that regulation of various brain functions by dopamine receptors is mainly accomplished through modulation of glutamatergic activity, particularly in striatal neurons [59]. Phasic dopamine signaling induced by drug administration activates the NAc and triggers synaptic changes involving NMDARs and AMPARs in response to glutamatergic projections from the prefrontal cortex and amygdala [60–63]. Additionally, repeated psychostimulant-induced sensitization requires activation of NMDARs by glutamate transmission [64]. Based on these evidences, we speculated that novel-m009C may function as a modulator of dopamine malfunction, secondarily altering NMDAR function, which underlies the development of METH addiction.

Novel-m009C is a regulator of METH reward but was found in the NAc of mice. Thus identifying a miRNA that is homologous to novel-m009C in humans is essential for its further application. Here, evolutionary analysis by MEGAX revealed that novel-m009C may be homologous to hsa-miR-604. We also validated that hsa-miR-604 could target Grin1 in *Homo sapiens*. The role of hsa-miR-604 in human diseases has been studied sporadically [65, 66]. To date, it has only been found that hsa-miR-604 expression is upregulated in the prefrontal cortices of subjects with alcohol use disorders postmortem [67]. The role of hsa-miR-604 in drug addiction has not yet been reported. Our findings are of great significance on the verification of the biological function of hsa-miR-604 and provided a clue that hsa-miR-604 may play a role in METH abuse. The differential changes in hsa-miR-604 expression in the postmortem brains and peripheral blood of METH abusers are worthy of in-depth research.

There were some limitations in the current study. Firstly, the downregulation of novel-m009C expression was across the NAc region, and the viruses induced overexpression or inhibition of novel-m009C expression was also throughout the whole NAc. Thus, it would be desirable to investigate the cell-specific expression patterns of novel-m009C in response to METH and examine if novel-m009C affects neuronal physiology as well as METH reward through regulating cell-specific target, such as modulating NMDAR-mediated synaptic plasticity through targeting cell-specific Grin1. Secondly, RIP-seq and RNA-seq data showed the involvement of a global gene network, not only Grin1, in the effects of novel-m009C on METH reward. Therefore, more screening and verification of the downstream target genes, and further investigation of the molecular and physiological mechanism by which the targets participate in the effects of novel-m009C are also warranted for uncovering the neurological mechanism of novel-m009C in METH abuse.

In summary, our study demonstrated that novel-m009C in the NAc is capable of regulating METH rewards and has clinical implications, as increased novel-m009C expression in the NAc might be a therapeutic strategy for combating METH addiction.

## Supplementary information

Supplementary information

Supplemental table 1

Supplemental table 2

## Data Availability

The RNA sequencing data analyzed during the current study are available from China National GeneBank DataBase (accession number: CNP0003085). Supplementary Information is available at MP’s website.
