# Supplementary material for: A novel microRNA, novel-m009C, regulates methamphetamine rewarding effects
Source: Mol Psychiatry. 2022 Jun 17;27(9):3885–97. doi: 10.1038/s41380-022-01651-2 (PMC9708597; doi:10.1038/s41380-022-01651-2)
Supplement: Supplementary file 1 — Supplementary information [file 41380_2022_1651_MOESM1_ESM.docx]

**Supplemental Experimental Procedure**

**Adeno-associated viral (AAV)**

An empty AAV vector (AAV-control) and an AAV vector carrying a scrambled sequence (AAV-scrambled) were selected as the corresponding controls for AAV-m009C and AAV-anti-m009C, respectively. AAV-control was used to evaluate whether the elements on the AAV vector alone (without any novel-m009C sequence) would have any non-specific and transgenic effects, while AAV-scrambled was used as the negative control for non-specific targeting effects. The ﬁnal virus preparations were titrated by real-time PCR, and the titres of the viral vectors were 5.33E+12 vg/ml for AAV-m009C and 2.5E+12 vg/ml for AAV-anti-m009C.

**Stereotaxic microinjection**

Each mouse was anaesthetized with isoflurane (4-5% for induction, 1-2% for maintenance) and placed in a stereotaxic frame (RWD, Shenzhen, China), and its skull was exposed and levelled. The AAVs were injected bilaterally into the NAc at the following coordinates: AP: +0.16 cm, ML: ± 0.26 cm, DV: -0.48 cm, 20° angle. The AAVs were microinjected at 0.6 μl per side at a speed of 0.2 μl/min. The microsyringe was left in place for 5 min after microinjection to allow diffusion of the AAV complexes. After surgery, injections of penicillin (0.02 mg/kg, i.p.) were given to the mice once daily for five consecutive days to prevent infection. Mice were housed with free access to food and water and given standard care.

**Conditioned place preference (CPP) test**

The CPP test was conducted after 1 week of habituation. On the day before the test (day 1), all mice not treated with any drug were allowed to explore the two compartments freely with the door open for 15 min. The mice were placed randomly in the white or black compartment to start the session. The time that each mouse spent in each compartment was recorded to determine the unconditioned preferences. The mice who cross less than 20 between the compartments were excluded. The following 8 days constituted the conditioning phase (days 2-9). On days 2, 4, 6 and 8, mice in one of the groups received one injection of saline and were confined in the black compartment for 40 min. On days 3, 5, 7 and 9, mice in the saline group received one injection of saline and were confined in the white compartment for 40 min; meanwhile, mice in the drug-treated group received an intraperitoneal injection of corresponding dose of drug (METH: 1.0 mg/kg, cocaine: 10.0 mg/kg or morphine: 10.0 mg/kg), and were confined in the white compartment for 40 min. Mice in the drug-treated group received saline and drug injections alternatively on two consecutive days, while mice in the saline group received saline injections in both compartments. For SPD and Arc pre-treatment, SPD or Arc was given 30 min prior to METH injection on days 3, 5, 7 and 9. The concentrations of SPD and Arc used for intraperitoneal injections were 30 mg/kg and 3.0 mg/kg, respectively. During the post-test period (day 10), all mice were allowed to explore the two compartments freely for 15 min without being subjected to injections, and the time that the mice spent in each compartment was recorded by an infrared monitoring system. The CPP score was calculated as the time that each mouse spent in the white compartment minus the time spent in the black compartment in the distinct test phases.

**Hyperlocomotion test**

Briefly, after 1 week of habituation, mice received saline injections intraperitoneally on day 1 and day 2 for adaptation to the injections. Then, the mice received either repeated drug (METH: 2.0 mg/kg, cocaine: 10.0 mg/kg, morphine: 5.0 mg/kg or MK-801: 1.0 mg/kg) or saline injections once daily for 5 consecutive days (day 3-day 7, development phase). After two drug-free days (day 8-day 9, transfer phase), mice in the drug-treated group received the same challenge dose of corresponding drug (METH, cocaine, morphine or MK-801), while mice in the saline group received one injection of saline on day 10 (expression phase). For SCH or RAC intervention, SCH, RAC or their corresponding vehicle-saline control was given 30 min prior to METH or saline injection on days 3-7 and day 10.

The horizontal locomotor activity test was performed in metal test chambers (43 cm × 43 cm × 43 cm), and the results were quantified by monitoring with a smart video tracking system. For assessing METH treatment, locomotor activity was monitored for 1 h before and after the METH or saline injections were performed on the treatment days, and the locomotor activity performed after the injections was recorded for data analysis. For assessing morphine treatment, locomotor activity of mice was monitored for 1 h before morphine or saline was injected on the treatment days. After the injection, locomotor activity was monitored and recorded for 90 min. For assessing cocaine and MK-801 treatment, locomotor activity was recorded for 1h before cocaine, MK-801 or saline injected on the treatment days. After the injection, locomotor activity was monitored and recorded for 30 min. The data from day 3, day 7 and day 10 were quantified for statistical analysis of the cocaine-, morphine- and MK-801-induced hyperlocomotion.

**Sucrose preference test**

On each test day, mice in the sucrose group were simultaneously presented with a bottle of water and a bottle of 1% (wt/vol) sucrose solution, while mice in the water group were presented with two bottles of water as the control. The bottles were weighed prior to being placed on the lid of each mouse’s home cage and reweighed to determine the amount of sucrose solution and water that had been consumed after 24 h. The positions of the bottles were changed every 12 h to ensure that the mice did not develop a preference for one side. Sucrose preference was calculated as the percentage of sucrose solution consumed relative to the total fluid intake: sucrose intake/(sucrose intake + water intake) × 100.

**Quantitative real-time PCR**

The following primers were used for amplification: novel-m009C precursor-forward, 5'- GCTGCGGTAGGAAGGATG-3' and reverse, 5'-GAGGGTGTGGGGTTGGGGAA-3'; Grin1-forward, 5'- GGCTGACTACCCGAATGTCCA-3' and reverse, 5'-TGTAGACGCGCATCATCTCAAAC-3'; and Gapdh-forward, 5'- TGTGTCCGTCGTGGATCTGA-3' and reverse, 5'- TTGCTGTTGAAGTCGCAGGAG-3'. For amplification of novel-m009C, miR-124-3p and miR-3078, a uni-miR qPCR primer (Takara, Japan) was used as the reverse primer, and the mature miRNA sequence was used as the forward primer.

**Western blot**

The NAc tissues were homogenized in ice-cold RIPA buffer with protease inhibitors. Homogenates were incubated on ice for 20 min and then centrifuged at 12,000 g for 5 min at 4 ℃. Supernatants were collected and the protein concentration in each sample was determined using a BCA protein assay kit (Pierce, Rockland, IL, USA). Protein samples were prepared with 5× protein loading buffer (HEART, China) and denatured at 95°C for 5 min. 15 μg of protein per sample was separated using 10% SDS-PAGE and transferred to polyvinylidene fluoride (PVDF) membrane (Millipore, USA). Then, the membranes were blocked with 5% (w/v) non-fat milk in 1 × Tris-buffered saline with 0.1% Tween-20 (TBST) at room temperature for 3 h. Blots were probed with the primary antibodies at 4℃ for overnight. The appropriate horseradish peroxidase-conjugated secondary antibodies were incubated for 1.5 h at room temperature. Signals were detected with an enhanced chemiluminescence assay kit (ECL Plus, Millipore Corporation, USA) and were visualized using ImageLab 1.46 (BioRad, USA). Primary antibody against Grin1(rabbit anti-NMDAR1, ab109182, Abcam) was at 1:1000 dilutions, and goat anti-rabbit lgG horseradish peroxidase-conjugated secondary antibody (Proteintech, USA) was at 1:2000 dilution. The results for western blot were analyzed using densitometry. Ratios of Grin1- to β-actin were calculated respectively for each sample. Saline was set at 1.

**RNA immunoprecipitation-sequencing (RIP-Seq) analysis**

For the Argonaute 2 (Ago2)-RIP assay, cell lysates were crosslinked with antibody (anti-Ago2, ab186733, Abcam)-bead complexes for 4 h at 4°C with rotation. Crosslinked RNA was then isolated. For the sequencing experiments, cDNA libraries of RIP samples were prepared after reverse transcription, adapter ligation and PCR enrichment. Illumina high-throughput sequencing was performed on the libraries. For sequencing data analysis, clean reads (50 bp single-end reads) were mapped to the mouse genome (mm9) after quality control, and unique mapped reads were used for further analysis. Peak calling was performed to locate the genomic regions enriched with reads. We then counted the peaks in 5′UTRs, CDSs and 3′UTRs and calculated the fold enrichment of reads in each region by dividing the number of reads in AAV-m009C samples by the number of reads in AAV-control samples and then log2 transforming the values. Regions with *P*<0.05 in the difference test were determined as regions with differential enrichment. Regions with differential enrichment were then annotated to genes.

**High-throughput RNA sequencing (RNA-Seq) analysis**

cDNA libraries of mRNA samples were prepared by poly(A) enrichment. The libraries were subjected to Illumina high-throughput sequencing. After quality control, the clean reads were mapped to the mouse genome (mm9). The mapped reads were assembled into transcripts and annotated to protein-coding genes using StringTie. FPKM (fragments per kilobase of transcript per million fragments mapped) was used for gene expression calculation. Differentially expressed genes were analysed by DESeq2. Data are presented as the mean value of three independent biological replicates. The fold change with respect to the normalized expression level was calculated as log2 (FPKM_AAV-m009C_/FPKM_AAV-control_). Genes with *P*<0.05 in the difference test were determined to be differentially expressed genes.

**Novel-m009C targetome analysis**

Genes (a total of 152) with increased fold enrichment in the 3’UTR (AAV-m009C vs AAV-control) were selected for subsequent analysis. Target site predictions were performed by RNAhybrid (v2.1.2)+svm_light (v6.01) and Miranda (v3.3a). RISC-enriched genes with target sites were finally compared to the downregulated genes identified by RNA-Seq.

**Novel-m009C phylogenetic analysis**

The miRNAs that were used for the “seed” sequencing blasting were annotated in miRBase (version 22.0). The phylogenetic tree (commonly known as an evolutionary tree or a phylogeny) between novel-m009C and miRNAs from other species was analysed by MEGA X software (version 10.1.8, USA).

**Ingenuity pathway analysis (IPA)**

Based on analysis with IPA software (version: 2018 summer), we identified significantly enriched (*P*<0.05) categories in a functional annotation chart. Then, genes in each category were annotated into secondary classifications. Z-scores were calculated to indicate the activation state of each functional classification. Further analysis was performed to explore the interconnection network between the genes and the secondary classifications according to the IPA database.

**Fluorescence in situ hybridization (FISH)**

Brain sections (4 μm) were dewaxed in water and rehydrated. Prehybridization was then performed at 65°C for 15 min. Sections were then hybridized overnight with fluorescently labelled riboprobes (1:150) in an incubator at 37°C. After hybridization, the sections were rinsed rigorously with SSC. Finally, after 8 min of incubation with DAPI, an anti-fluorescence quenching sealer was dripped onto the slices for sealing. The stained sections were examined under an inverted fluorescence microscope (Nikon Eclipse TI-SR, Japan) and imaged (Nikon DS-U3, Japan). Novel-m009C antisense probe: 5'-CCGCAUCCUUCCUACCGCAGCU-3'. Negative control probe: 5'-GTGTAACACGTCTATACGCCCA-3'. FISH data for novel-m009C was analyzed using Image J (National Institute of Health, USA) and mean fluorescence intensity (integrated density / area) comparing METH and saline-treated group was calculated for statistical analysis.

**Dual-luciferase reporter assay**

HEK-293T cells were co-transfected with the pMIR reporter vector containing the Grin1 3′UTR with the predicted novel-m009C or hsa-miR-604 binding site or the corresponding mutated binding site (transcript NM_008169.3 for novel-m009C; transcript NM_007327.4 for hsa-miR-604) and novel-m009C or hsa-miR-604 mimics or the corresponding control mimics. After transfection for 48 h, dual-luciferase reporter assays were performed using a luminometer (Spark 10M, TECAN) according to the manufacturer’s protocol. The ratio of firefly luciferase activity to Renilla luciferase activity was calculated.

**Supplemental Figures**

**Supplemental Figure 1.**

**
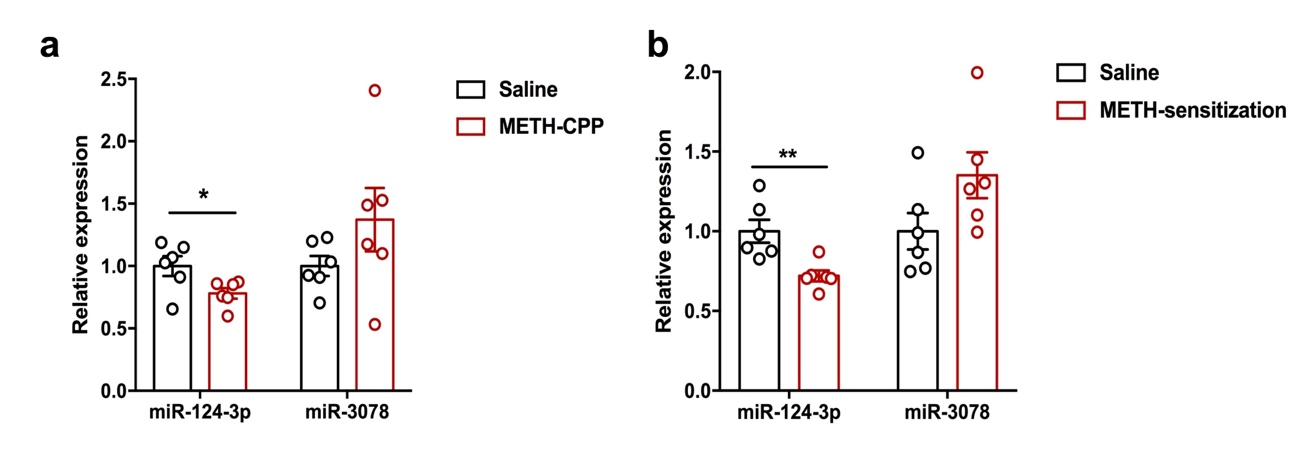
**

**Differential expressions of miR-124-3p and miR-3078 in the NAc of mice following METH-CPP and sensitization tests. a.** Significant decrease in the expression of miR-124-3p but not miR-3078 in the NAc of mice subjected to the METH-induced CPP test. miR-124-3p fold change: t_(10)_ = 2.429; miR-3078: t_(10)_ = 1.397; **P*<0.05; n=6. **b.** Significant decrease in the expression of miR-124-3p but not miR-3078 in the NAc of mice subjected to the METH-induced hyperlocomotion test. miR-124-3p fold change: t_(10)_ = 3.489; miR-3078: t_(10)_ = 1.909; ***P*<0.01; n=6. All values are presented as the mean ± SEM.

**Supplemental Figure 2.**

**
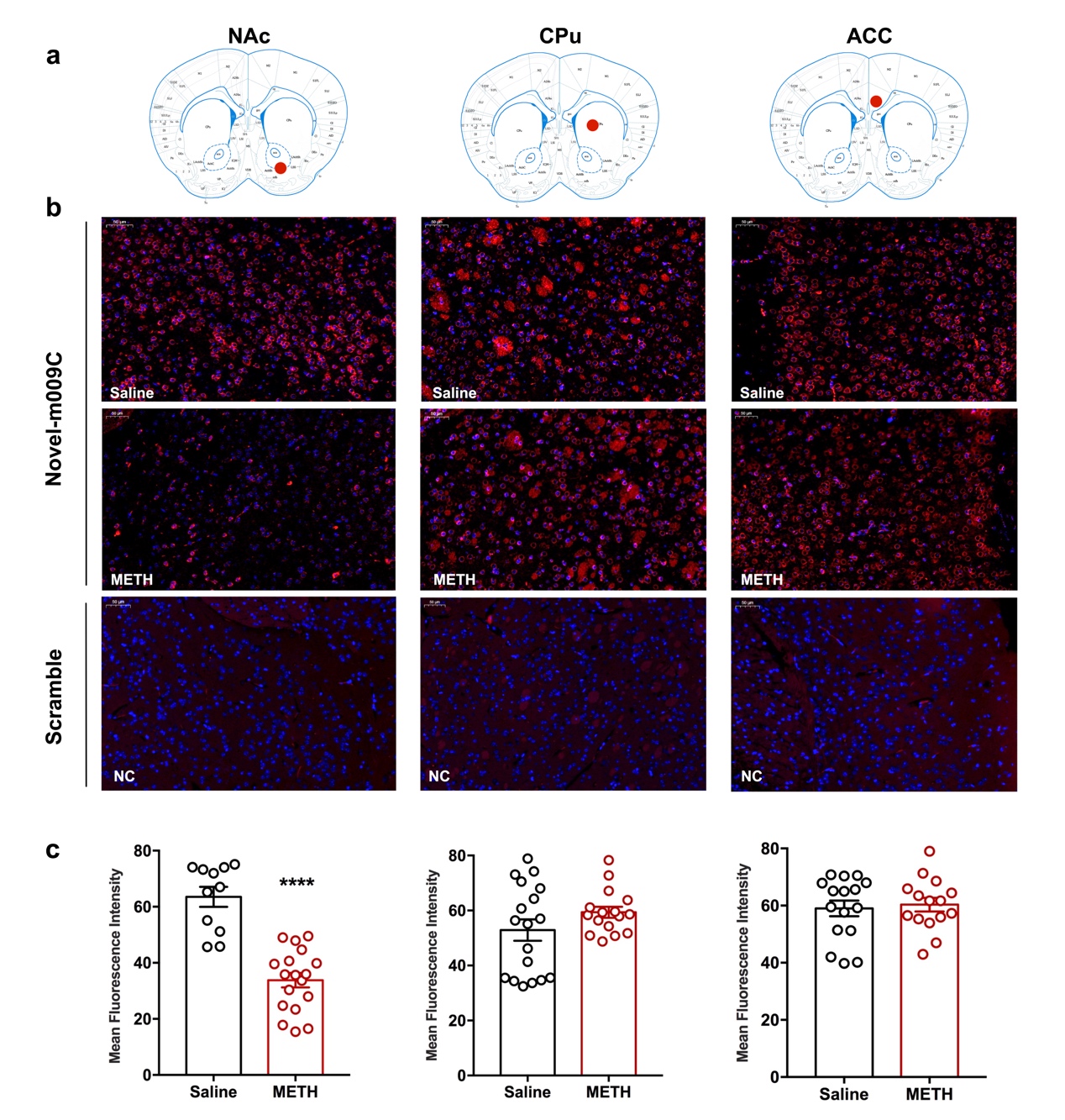
**

**Differential expressions of novel-m009C in the NAc, CPu and ACC in METH versus saline-treated mice by FISH assay. a.** Schematic diagram of brain regions in the NAc (left), CPu (middle), and ACC (right). **b.** Fluorescence revealed the novel-m009C (red) was expressed in the cytoplasm of cells in the NAc (left), CPu (middle), and ACC (right) in saline- (first row) and METH-treated mice (second row); The third row showed the negative control (NC) for novel-m009C in the NAc (left), CPu (middle), ACC (right) of mice; riboprobe with a scrambled sequence was used as the negative control for novel-m009C. **c.** Statistical analysis of the mean fluorescence intensity of novel-m009C in the NAc (left), CPu (middle), and ACC (right) in METH versus saline-treated mice. Independent t-test. NAc (left): t_(27)_ = 6.686; CPu (middle): t_(32)_ = 1.435; ACC (right): t_(29)_ = 0.3597. *****P*<0.0001. Novel-m009C was stained red; nuclei, stained with DAPI (blue); Scale bar: 50 μm. All values are presented as the mean ± SEM.

**Supplemental Figure 3**

**
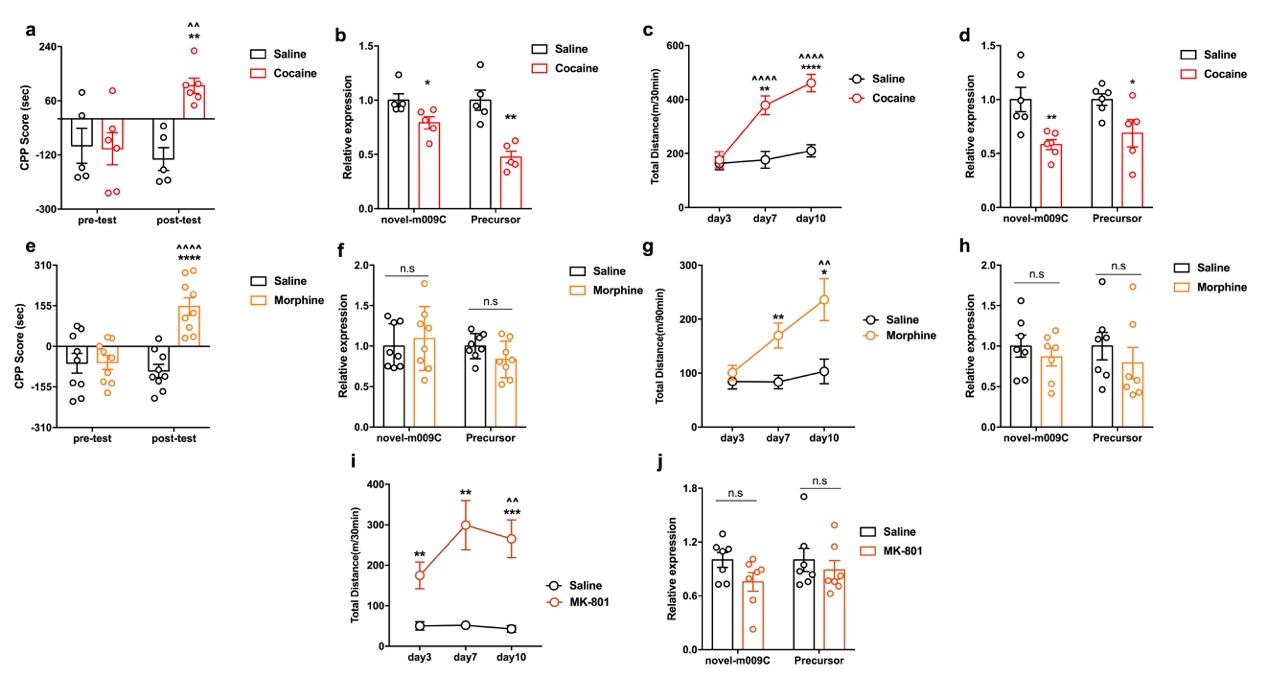
**

**The differential expressions of** **novel-m009C in the NAc of mice in response to other psychiatric stimuli-Cocaine, morphine and MK-801. a.** Cocaine-induced CPP in mice. Two-way ANOVA. Cocaine: F_(1,18)_=7.674, *P* < 0.05; test: F_(1,18)_=3.344, *P* = 0.0841; cocaine× test: F_(1,18)_=7.844, *P* < 0.05; ***P*<0.01, different from the post-test score of the saline group; ^^*P*<0.01, different from the pre-test score of the same group; n=5-6. **b.** Expression of novel-m009C and its precursor in the NAc of mice after cocaine-induced CPP. Independent t-test. Novel-m009C fold change: t _(8)_ = 2.529; precursor fold change: t_(8)_ = 4.862; **P* < 0.05, ***P* < 0.01; n=5. **c.** Cocaine-induced locomotor sensitization in mice. One-way repeated measures ANOVA. Cocaine: F _(1, 10)_ = 16.149, *P* < 0.01; day: F _(2, 20)_ = 85.626, *P* < 0.001; cocaine × day: F _(2, 20)_ = 48.020, *P* < 0.001. ***P*<0.01, *****P*<0.0001, different from the saline group; ^^^^*P* < 0.0001, different from the same group on day 3; n=6. **d.** Expression of novel-m009C and its precursor in the NAc of mice after cocaine sensitization. Independent t-test. Novel-m009C fold change: t_(10)_ = 3.415; precursor fold change: t_(9)_ = 2.439; ***P*<0.01, **P*<0.05; n=5-6. **e.** Morphine-induced CPP in mice. Two-way ANOVA. Morphine: F_(1, 32)_ = 16.21, *P* < 0.001; test: F_(1, 32)_ = 8.842, *P* < 0.01; morphine × test: F_(1, 32)_ = 15.38, *P* < 0.001; *****P* < 0.0001, different from the post-test score of the saline group; ^^^^ *P*<0.0001, different from the pre-test score of the same group; n=9. **f.** Expression of novel-m009C and its precursor in the NAc of mice after morphine-induced CPP. Independent t-test. Novel-m009C fold change: t_(14)_ = 0.5463; precursor fold change: t_(14)_ = 1.699; n.s.: *P* > 0.05; n=8. **g.** Morphine-induced locomotor sensitization in mice. One-way repeated measures ANOVA. Morphine: F _(1, 14)_ = 9.893, *P* < 0.01; day: F _(2, 28)_ = 9.717, *P* < 0.01; morphine × day: F _(2, 28)_ = 5.614, *P* < 0.01. **P*<0.05, ***P* < 0.01, different from the saline group; ^^*P* < 0.01, different from the same group on day 3; n=8. **h.** Expression of novel-m009C and its precursor in the NAc of mice after morphine sensitization. Independent t-test. Novel-m009C fold change: t_(12)_ = 0.7727; precursor fold change: t_(12)_ = 0.8204; n.s.: *P* > 0.05; n=7. **i.** MK-801 induced hyperlocomotion. One-way repeated measures ANOVA. MK-801: F _(1, 14)_ = 21.174, *P* < 0.0001; day: F _(2, 28)_ = 5.029, *P* < 0.05; MK-801 × day: F _(2, 28)_ = 5.169, *P* < 0.05. ***P* < 0.01, ****P*<0.001, different from the saline group; ^^*P* < 0.01, different from the same group on day 3; n=8.  **j.** Expression of novel-m009C and its precursor in the NAc of mice after MK-801 treatment. Independent t-test. Novel-m009C fold change: t_(12)_=1.842; precursor fold change: t_(12 )_ = 0.6695; n.s.: *P* > 0.05; n=7. All values are presented as the mean ± SEM.

**Supplemental Figure 4.**


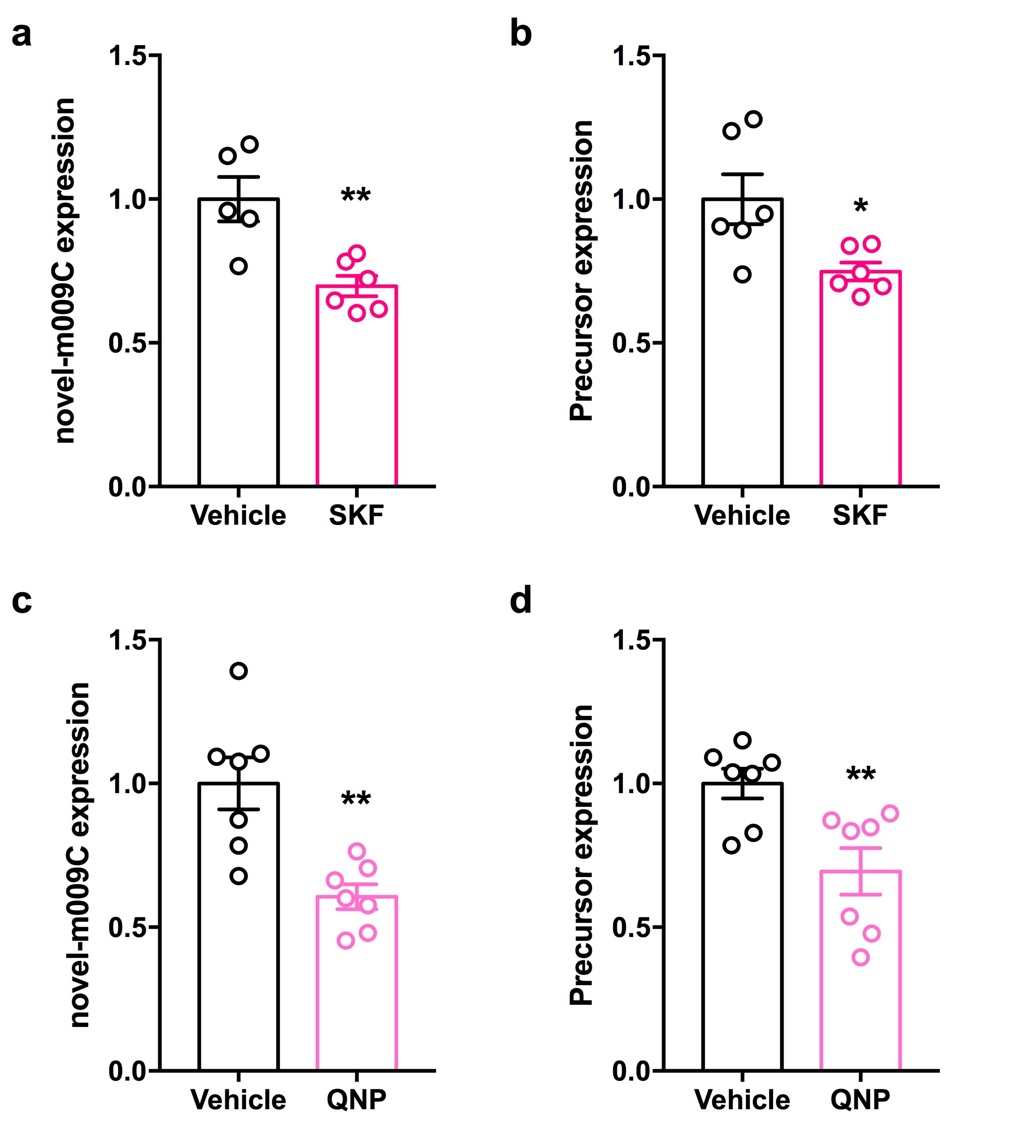


**D1R and D2R agonists induced significantly decreases in the expression of novel-m009C and its precursor in the NAc of mice.**  **a and b.** D1R agonist SKF decreased the expression of novel-m009C (a) and its precursor (b). Independent t-test. Novel-m009C fold change: t_(9)_=3.774; precursor fold change: t_(10)_=2.731; ***P*<0.01, **P*<0.05; n=5-6. c and d. D2R agonist QNP decreased the expression of novel-m009C (c) and its precursor (d). Independent t-test. Novel-m009C fold change: t_(12)_=3.927; precursor fold change: t_(12)_=3.174; ***P*<0.01; n=7. SKF: SKF-38393, 5mg/kg, i.p; QNP: Quinpirole, 0.5mg/kg, i.p. All values are presented as the mean ± SEM.

**Supplemental Figure 5.**

**
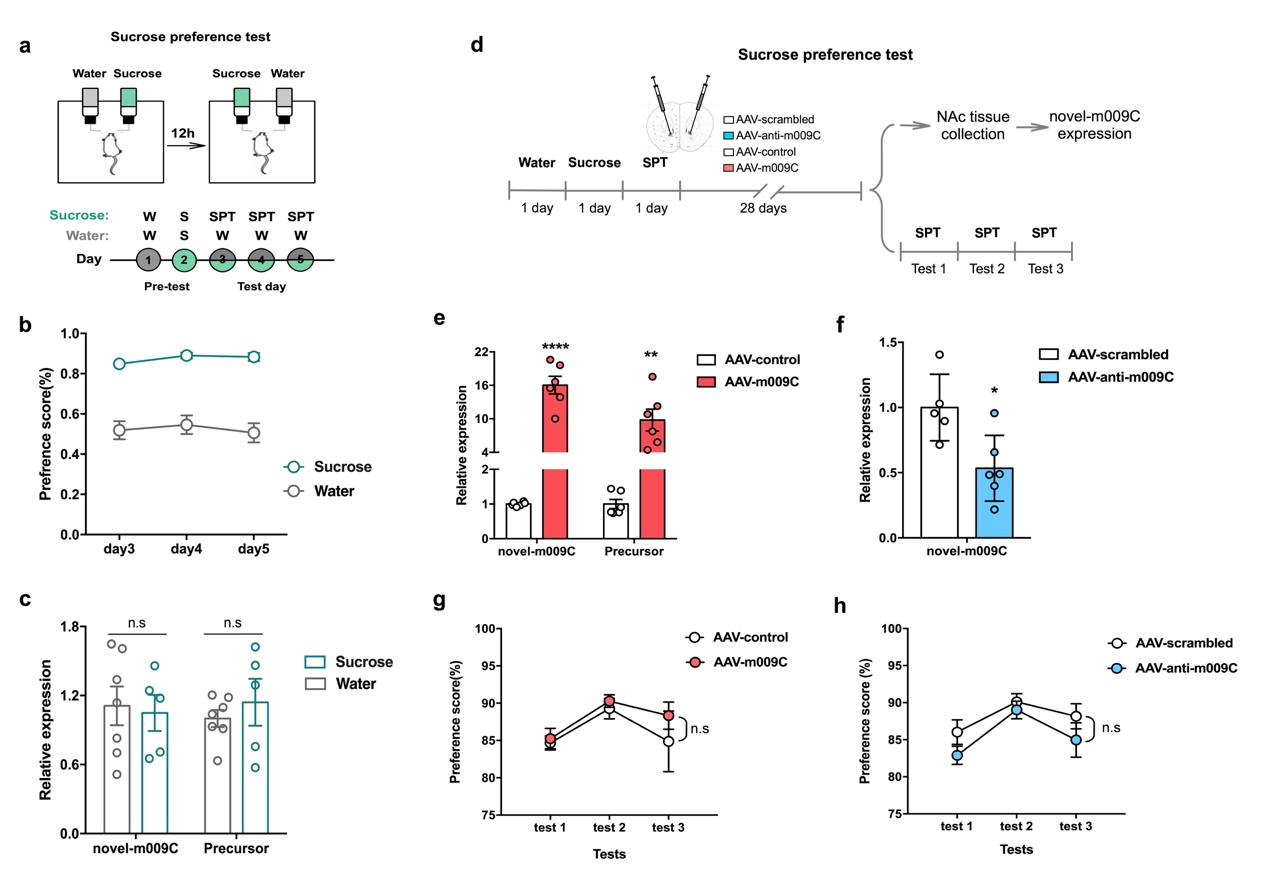
**

**Novel-m009C in the NAc did not regulate preference for sucrose in mice. a.** Timeline of the SPT. **b.** The mice showed a natural preference for sucrose, since the percent of sucrose consumption is more than 60%; n=7. **c.** The expression of novel-m009C and its precursor in the NAc of mice did not change in response to sucrose exposure. NAc samples were collected after the last trial of the SPT. Independent t-test. Novel-m009C fold change: t_(10)_=0.2591; precursor fold change: t_(10)_ = 0.7402; n.s.: *P*>0.05; n=5-7. **d.** Timeline of AAV injection and SPT. **e.** The expression of novel-m009C and its precursor was substantially upregulated in the NAc of AAV-m009C-injected mice. Independent t-test. Novel-m009C fold change: t_(10)_=9.498; precursor fold change: t_(10)_=4.453. ***P*<0.01, *****P*<0.0001; n = 6. **f.** The expression of novel-m009C was significantly downregulated in the NAc of AAV-anti-m009C-injected mice. Independent t-test: t_(9)_ = 3.041; **P* < 0.05; n = 5-6. **g-h.** Overexpression (g) or inhibition (h) of novel-m009C in the NAc did not change the preference of mice for sucrose. One-way repeated measures ANOVA. Overexpression: virus: F_(1, 14)_=1.035, *P* = 0.326; test: F_(2, 28)_=2.824, *P* = 0.076; virus × test: F_(2, 28)_=0.281, *P* = 0.757; n.s.: *P*>0.05; n = 8; Inhibition: virus: F_(1, 11)_ = 0.902, *P* = 0.363; test: F_(2, 22)_ = 4.099, *P* = 0.031; virus × test: F_(2, 22)_ = 0.072, *P* = 0.931; n.s.: *P* > 0.05; n=6-7. All values are presented as the mean ± SEM.

**Supplemental Figure 6.**


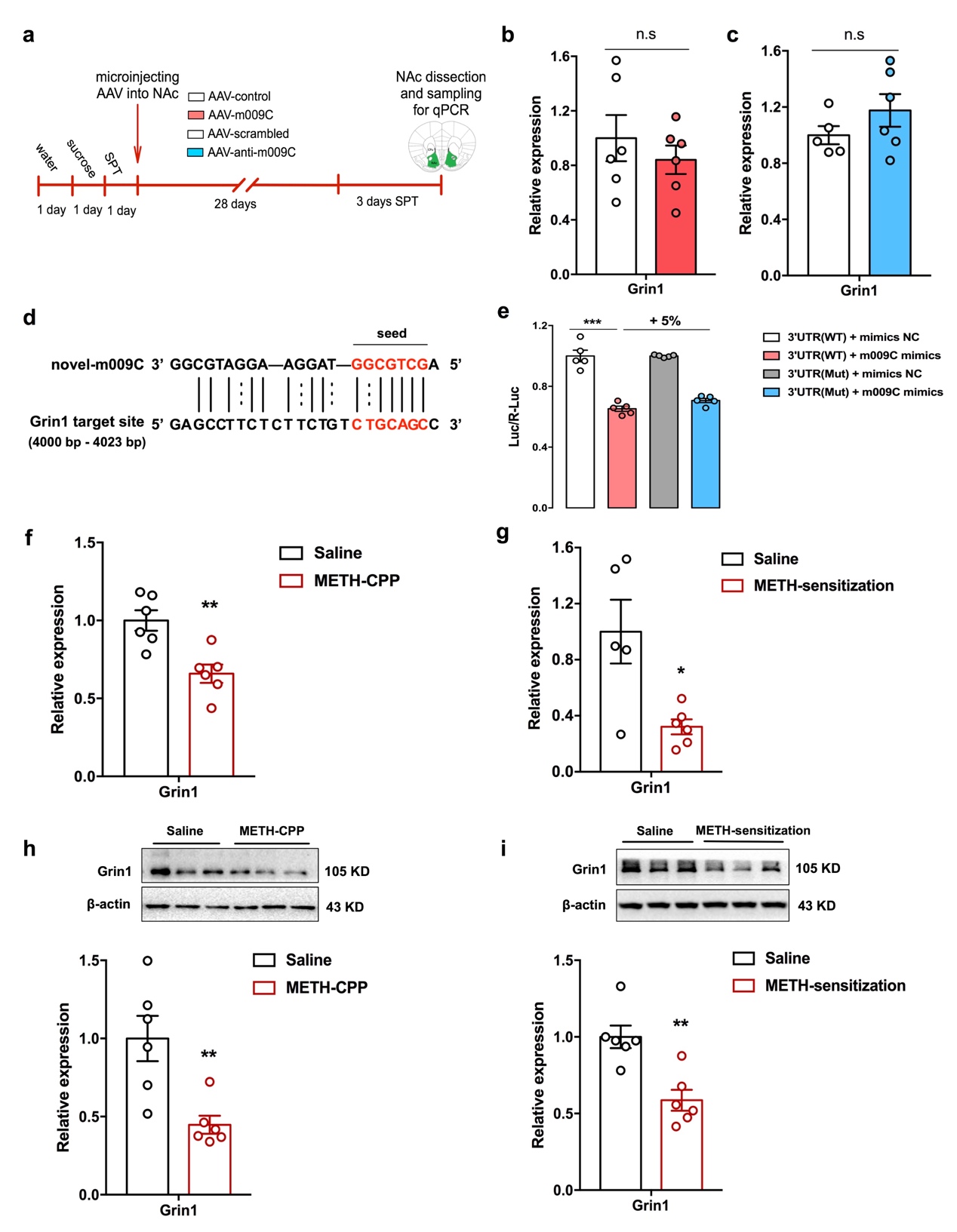


**Grin1 was not changed in the NAc of mice whether novel-m009C over-expressed or inhibited following the SPT, but was significantly changed in response to METH. a.** Procedure for measuring Grin1 expression in the NAc of AAV-injected mice following 3 days of the SPT. NAc samples were collected after the final trial of the SPT. **b and c.** Grin1 expression in the NAc was significantly changed in neither the AAV-m009C group (b) nor AAV-anti-m009C group (c) following the SPT. Independent t-test. Overexpression: t _(10)_ = 0.7974; inhibition: t _(9)_ = 1.248; n.s.: *P*>0.05; n=5-6. **d.** Target site between novel-m009C and Grin1 3’UTR. The “seed” sequence is shown in red. **e.** A dual luciferase reporter assay was used to assess the targeting of Grin1 by novel-m009C in mice. The groups were compared by analysing the L-Luc/R-Luc ratio. Independent t-test: t_(8)_ = 8.349, ****P*<0.001, different from the 3’UTR (WT) + mimics NC group; n=5. 3’UTR (WT): vector expressing luciferase the WT Grin1 3’UTR; mimics NC: scrambled-control mimics; m009C mimics: novel-m009C mimics; 3’UTR (Mut): vector expressing luciferase and the mutant Grin1 3’UTR. **f and g.** The mRNA level of Grin1 expression was significantly reduced in the NAc of METH-CPP (f) and METH-sensitization (g) mice, respectively. Independent t-test. Grin1 fold change: CPP: t _(10)_=3.878, sensitization: t _(9)_=3.178; ***P*<0.01, **P*<0.05; n=5-6. **h and i.** The protein level of Grin1 expression was significantly reduced in the NAc of METH-CPP (h) and METH-sensitization (i) mice, respectively. Independent t-test. Grin1 fold change: CPP: t_(10)_=3.533, sensitization: t_(10)_=4.125; ***P*<0.01; n=6. All values are presented as the mean ± SEM.

**Supplemental Figure 7.**

**
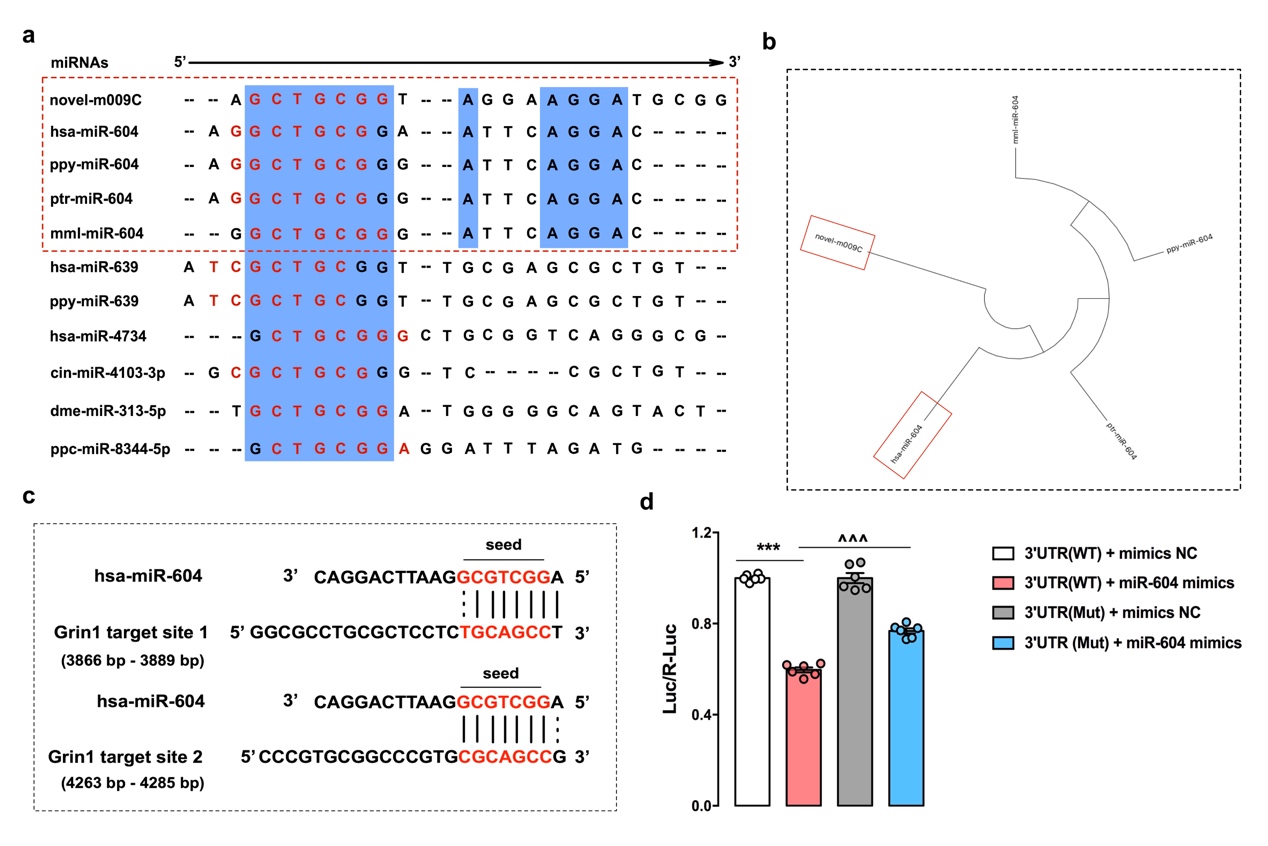
**

**Novel-m009C may be homologous to hsa-miR-604.** **a.** Sequence analysis of novel-m009C and miRNAs from other species by blast. Red: the “seed” sequence of miRNA; blue: the same sequence in novel-m009C and miRNAs. Red dotted box: the sequence of novel-m009C showed the greatest similarity to primate-specific miRNA-miR-604. **b.** Phylogenetic tree between novel-m009C and primate-derived miR-604, and hsa-miR-604 and novel-m009C are the closest in evolutionary relationship. **c.** Two target sites between hsa-miR-604 and the Grin1 3’UTR. The “seed” sequence is shown in red. **d.** A dual luciferase reporter assay was used to assess the targeting of Grin1 by hsa-miR-604 in *Homo sapiens*. Comparisons of the L-Luc/R-Luc ratio among groups. Independent t-test: t_1 (10)_ = 32.36, t_2 (10)_ = 10.77; ****P*<0.001, different from the 3’UTR (WT) + mimics NC group; ^^^*P*<0.001, different from the 3’UTR (WT) + miR-604 mimics; n=6. 3’UTR (WT): vector expressing luciferase and the WT Grin1 3’UTR; mimics NC: scrambled-control mimics; miR-604 mimics: hsa-miR-604 mimics. 3’UTR (Mut): virus expressing luciferase and the mutant Grin1 3’UTR. All values are presented as the mean ± SEM.
